# Two *Phytophthora* effectors mitigate plant immunity by manipulating intracellular pH through interaction with V-ATPase in potato

**DOI:** 10.1186/s43897-025-00184-w

**Published:** 2026-01-09

**Authors:** Xiaojing Xue, Xiao Chen, Xin Wang, Tao Wang, Rui Liao, Haifeng Liu, Changxiang Zhu, Jonathan D. G. Jones, Zhaohui Chu

**Affiliations:** 1 State Key Laboratory of Hybrid Rice, Hubei Provincial Research Center for Basic Biological Sciences, College of Life Sciences, Wuhan University, Wuhan, 430072 China; 2 Hubei Hongshan Laboratory, College of Life Sciences, Wuhan University, Wuhan, 430072 China; 3 State Key Laboratory of Wheat Improvement, College of Agronomy, Shandong Agricultural University, Taian, 271018 China; 4 The Sainsbury Laboratory, Norwich Research Park, Norwich, United Kingdom

**Keywords:** V-ATPase, Cytoplasmic pH, Disease resistance, Late blight, Oomycetes, RxLR effector, NLRs

## Abstract

Plant V-ATPase serves as a primary active proton pump of the endomembrane system and is crucial for the stress response. However, the role of the C subunit of V-ATPase (VHA-C) in effector-triggered immunity remains poorly understood. Here, we reveal that *Phytophthora infestans* evolved a pair of RxLR effectors, AL3 and Avr2, which are expressed sequentially and both target the host VHA-C (StATP6V1C1) and StBSL1. In the early stage of *P. infestan*s infection, AL3 promotes the assembly of StATP6V1C1 with subunits G and E, leading to increased V-ATPase activity and cytoplasmic acidification. Subsequently, Avr2 inhibits the StWNK10-catalyzed Ser-261 phosphorylation of StATP6V1C1, thereby retarding V-ATPase activity and causing intracellular alkalinization. In cultivars absence of two immune receptors, this pH shift facilitates the interactions of the two effectors with downstream susceptibility factors of StBSL1 at various stages of infection, which may promote the onset and development of the disease. As coping strategy, plants independently evolve two NLRs, R2 and Rpi-mcq1, guard both StATP6V1C1 and StBSLs to perceive effectors thereby mitigating the risk of late blight. Our findings establish a new arms race battlefield between plants and oomycetes, highlighting the role of intracellular pH homeostasis in both effector-triggered susceptibility (ETS) and effector-triggered immunity (ETI).

**Supplementary Information:**

The online version contains supplementary material available at 10.1186/s43897-025-00184-w.

## Core

This study reveals a novel *Phytophthora infestans* RxLR effectors AL3, coordinated with Avr2, targeted and bidirectionally regulated the assembly of potato V-ATPase to manipulate the intracellular pH during infection. In the presence of the R2 or Rpi-mcq1 receptor, StATP6V1C1 plays a crucial role in the recognition between R2/Rpi-mcq1 and Avr2/AL3, mediating a decoy model of multiple R-Avr recognitions.

## Gene & accession numbers

The sequence data used in this study can be found in the GenBank data libraries under the following accession numbers: PITG_22870 (XM_002902940), PITG_23008 (XM_002899560), StATP6V1C1 (XM_006363911), StBSL1 (NM_001318640), StBSL2/3-like (NM_001288309), StATP6V1G (XM_015308645), StATP6V1E (XM_006343836), StWNK10 (XM_006343735), AtWNK8 (AT5G41990).

## Introduction

pH fluctuations serve as signals that regulate various physiological activities in plants, including immune responses (Li et al. 2022). The acidic extracellular space of the apoplast is essential for numerous plant–microbe interactions. Apoplast alkalinization is a hallmark of the plant pathogen-associated molecular pattern (PAMP)-triggered immune (PTI) response. For instance, Pep1, flg22, and elf18 can rapidly alkalinize cell suspension media (Felix et al. 1999; Yamaguchi et al. 2006; Zipfel et al. 2006). PTI increases the apoplastic pH in Arabidopsis's root apical meristem (RAM) region, promoting the binding of Pep1 to PEPR and strengthening root immunity (Liu et al. 2022). Additionally, acidification has been recognized as a common intracellular response to elicitors in plant cells and is linked to the expression of various defense-related genes (Mathieu et al. 1996; Lapous et al. 1998; Liu et al. 2022). For example, a decrease in intracellular pH in barley coleoptiles has been associated with resistance against Blumeria graminis (Yamaoka et al. 2000). However, little is known about how intracellular pH fluctuates and its role in plant–microbe interactions.

Plants achieve precise pH regulation in each subcellular compartment by modulating the activity of at least four distinct types of proton pumps: phosphorylated intermediate-type ATPases (P-ATPase), vacuole-type proton pumps (including V-PPase and V-ATPase), FOF1 -type ATPases (F-ATPase), and ATP-binding cassette transporters (ABC transporters) (Gaxiola et al. 2007; Li et al. 2022). The P-ATPase, V-PPase, and ABC transporters consist of a single polypeptide, while the V-ATPase (VHA) and F-ATPases are complex arrays of subunits (Gaxiola et al. 2007; Seidel et al. 2022). In general, P-ATPase pumps H^+^ from the cytoplasm across the cell membrane, whereas V-PPase and V-ATPase acidify the intracellular compartments, including the vacuole, and F-ATPase is anchored to the mitochondrion and chloroplast. Many previous reports revealed that the earliest cellular responses to pathogen infection are regulated by plasma membrane-localized enzymes and ion channels, including P-ATPase. The pathogen Pseudomonas fuscovaginae was found to produce phytotoxic lipodepsipeptides, which have dual effects on rice P-ATPase activity: stimulating ATPase activity at low concentrations in a detergent-like manner but inhibiting the enzyme at relatively high concentrations (Batoko et al. 1998). Additionally, the *Phytophthora capsici *RxLR effector CRISIS2 triggers cell death by suppressing P-ATPase in the host plant (Seo et al. 2023). Moreover, the immune-negative regulatory protein RIN4 interacts with the P-ATPases AHA1 and AHA2 to increase stomatal opening during bacterial invasion of Arabidopsis (Liu et al. 2009). Interestingly, the Avr5 effector of Cladosporium fulvum is recognized by the cognate R protein Cf5 in tomato, resulting in the activation of P-ATPase, followed by acidification of the extracellular matrix (Veraestrella et al. 1994). Furthermore, apoplastic acidification mediated by an activated P-ATPase is observed during the interaction between barley Mla3 and the AvrMla3 effector of the powdery mildew fungus B. graminis (Zhou et al. 2000). The evidence above shows that all stages of plant immunity are accompanied by fluctuations in P-ATPase activity and cellular pH. Phosphorylation has been proposed as a regulatory mechanism for P-ATPase activity. For example, the phosphorylation of Thr947 and Thr881 increases, whereas the phosphorylation of Ser899 and Ser931 inhibits the P-ATPase activity of AHA2.

V-ATPase is a heterooligomeric enzyme composed of 14 subunits that are organized into a cytoplasmic domain (V1) and a proton translocation transmembrane domain (V0). The assembly of V1-V0 and the formation of the proton channel require V-ATPase subunit C (ATP6V1C1, VHA-C) (Vasanthakumar et al. 2020; Li et al. 2022; Seidel et al. 2022; Wang et al. 2023). V-ATPase is located primarily in the tonoplast and is distributed in the endoplasmic reticulum, Golgi apparatus, vesicles, and other secretory system membranes (Vasanthakumar et al. 2020; Seidel et al. 2022). It is mainly responsible for maintaining cytoplasmic pH homeostasis and regulating endocytosis, ROS signaling, and secretory transport. In animal immunity, V-ATPase plays a crucial role in maintaining the pH homeostasis in lysosomes to activate the immune response and in cytokine trafficking and secretion during inflammation, and making it an attractive target for pathogen attack (Hu et al. 2023; Pu et al. 2024). For example, a Salmonella type three secretion system (T3SS) effector, SopF, specifically ADP-ribosylates Gln124 of ATP6V0C in the V-ATPase to hinder its recruitment of ATG16L1, potently blocking antibacterial autophagy (xenophagy) (Xu et al. 2019). In contrast to P-ATPase, there are fewer studies on the function of plant V-ATPase in the immune system. Recent research suggests that V-ATPase is involved in plant antiviral defense. Barley stripe mosaic virus (BSMV) replicase γα interact with V-ATPase catalytic subunit B2 (VHA-B2) to disrupt the interaction between VHA-B2 and E, to suppress vacuolar acidification and to facilitate virus infection (Yang et al. 2022). To combat infection by the RNA virus TuMV, the host ATP6V0C is upregulated, which can specifically target and degrade the proviral factor FAD4, inhibiting viral infection (Fang et al. 2024). Although there is evidence that some interacting proteins that interact with VHA, such as 14–3-3 and WNK8 (Hong-Hermesdorf et al. 2006), may regulate enzyme activity, the specific regulatory molecular mechanisms involved remain to be elucidated. Other types of plant H^+^ pumps are also attracting attention. For example, a putative ABC transporter, Lr34, has been shown to provide durable resistance to multiple fungal pathogens in wheat (Krattinger et al. 2009).

*Phytophthora infestans* is a notorious oomycete pathogen that can cause potato late blight and possesses over 500 candidate RxLR effectors (Haas et al. 2009). Diverse effectors are delivered into host cells and interact with a broad set of cellular targets associated with various biological processes (Petre et al. 2021). Effector-target interactions require a specific pH environment. For example, the EPIC (*P. infestans*)-C14 (*Solanum lycopersicum*) interaction occurs across a wide pH range compared to the AVR2 effector (C. fulvum), which preferentially inhibits the tomato defense proteases PIP1 and RCR3 at an apoplastic pH (Kaschani et al. 2010). Avr2 is the most extensively studied *P. infestans* RxLR effector and is recognized by two distinct nucleotide-binding leucine-rich repeat receptors (NLRs), R2 and Rpi-mcq1 (Aguilera-Galvez et al. 2019). Avr2 targets the brassinosteroid (BR) signal transduction component BSL1 and mediates the interaction of BSL1 with R2 in planta, possibly through the formation of a ternary complex (Saunders et al. 2012). Avr2 suppresses plant immunity by upregulating a BR-responsive bHLH transcription factor, exploiting antagonistic crosstalk between BR signaling and innate immunity (Turnbull et al. 2017). Interestingly, VHA-a2 and VHA-a3 interact with BZR1 and negatively regulate BR signaling in Arabidoposis (Jiang et al. 2023). Emerging evidence suggests that BSL2/3 is necessary for Rpi-mcq1-Avr2 recognition (Turnbull et al. 2019; Wang et al. 2021). This means that one effector may be recognized by multiple R proteins through different intermediate proteins, even within the same BSL family. In addition to Avr2, three other Avr2 family effectors, PexRd11 (PITG_13930), PITG_21949, and PITG_21645, can also be recognized by R2, indicating that one R protein may recognize different effectors by monitoring target proteins (Saunders et al. 2012). To date, Avr2-BSL1/BSL2/3-(R2/Rpi-mcq1) remains the most complex R-Avr recognition model revealed in the Solanaceae-oomycete pathogen interaction system.

In this study, we found that *P. infestans* sequentially secretes two effectors, AL3 (PITG_23008) and Avr2, which target the C subunit of V-ATPase. This manipulation of host intracellular pH facilitates the targeting of the for easy to target downstream susceptibility factor StBSL1, ultimately leading to host susceptibility. Two independently evolved NLRs, R2 and Rpi-mcq1, guard both StBSL1 and StATP6V1C1 to recognize Avr2 and AL3, thereby enhancing host immunity. Our results establish a new battlefield for the arms race in terms of intracellular pH homeostasis, and also point out the importance of V-ATPase in both Effector-Triggered Susceptibility (ETS) and Effector-Triggered Immunity (ETI).

## Results

### Both Avr2 and AL3 can be recognized by R2 and Rpi-mcq1

Previously, PiAvr2 was recognized by two NLRs, R2 and Rpi-mcq1, which are mediated by BSL1 and BSL2/3, respectively (Aguilera-Galvez et al. 2019; Wang et al. 2021). In the T30-4 genome, the PiAvr2/PEXRD11 members comprise 13 RxLR effectors encoded by 18 genes, with shared amino acid sequence identities ranging from 28 to 98% (Fig. S1a,b). PiAvr2 and PITG_22870, PITG_08278 and PITG_20025, PITG_21645 and PITG_13956, PITG_23009 and PITG_23008 are present as double copies.

**Fig. 1:**
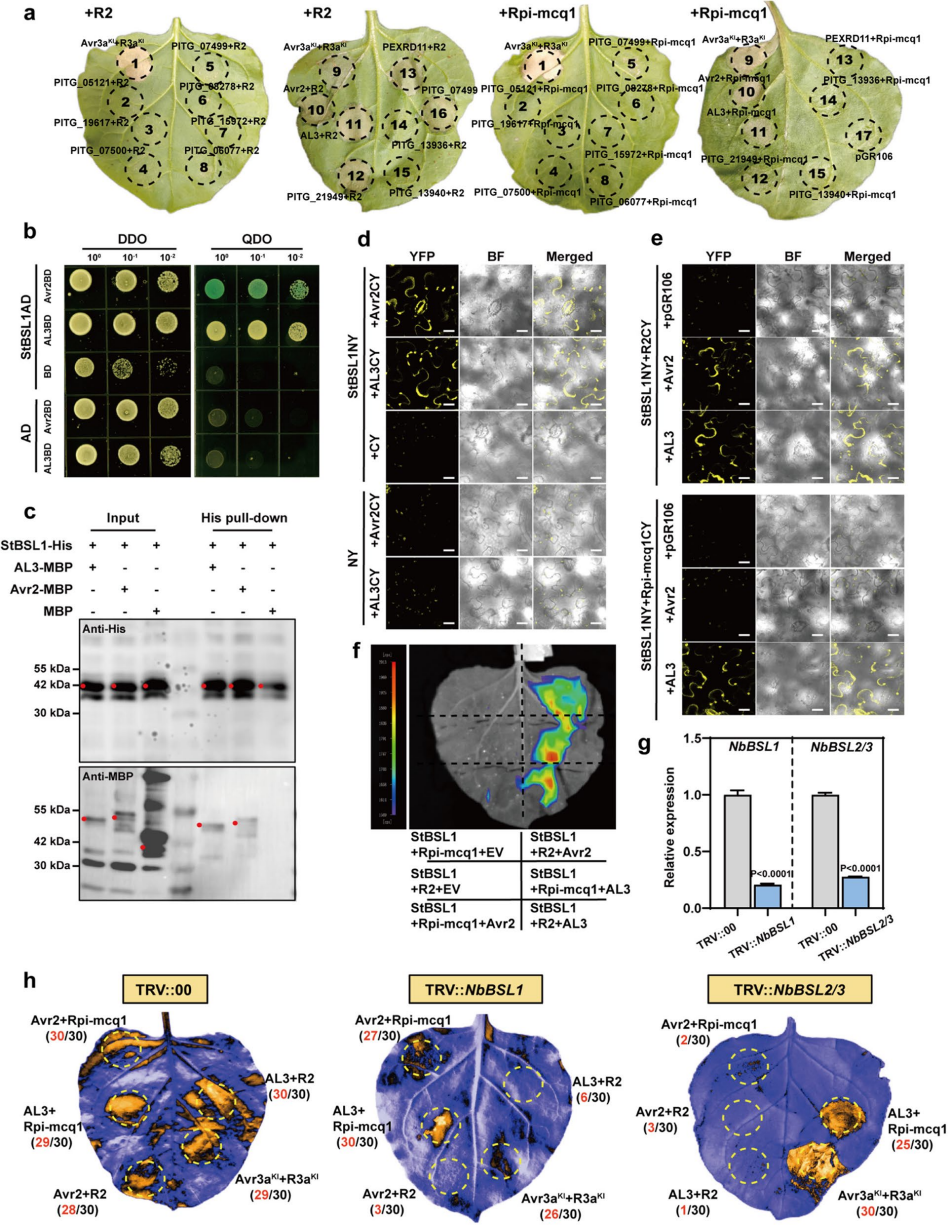
AL3 is recognized by Rpi-mcq1 in a way that does not depend on *BSLs.* **a**, Both Avr2 and AL3 can be recognized by R2 and Rpi-mcq1. Co-expression of Avr2 family members with R2 or Rpi-mcq1 was conducted in six-week-old *N. benthamiana* leaves. The hypersensitive response (HR) was observed 7 days after infiltration (*n* = 10). The following combinations were tested: 1. Avr3a^KI^ + R3a^KI^; 2. PITG_05121; 3. PITG_19617; 4. PITG_07500; 5. PITG_07499; 6. PITG_08278; 7. PITG_15972; 8. PITG_06077; 9. Avr3a^KI^ + R3a^KI^; 10. Avr2; 11. AL3; 12. PITG_21949; 13. PEXRD11; 14. PITG_13936; 15. PITG_13940; 16. PITG_07499 (only); 17. pGR106 (only). **b**-**d**, Avr2 and AL3 proteins interacted with the phosphatase domain of StBSL1 (amino acids 519–878). **e**–**f**, Avr2/AL3 induced interactions between StBSL1 and R2/Rpi-mcq1, with scale bars representing 20 µm. The experimental groups included the empty vector (EV, pGR106), Avr2/AL3 (pGR106-Avr2/AL3), StBSL1 (StBSL1-cLUC), and R2/Rpi-mcq1 (R2/Rpi-mcq1-nLUC), *n* = 8, t-test (*P* < 0.05). **g**-**h**, Rpi-mcq1-AL3 recognition was independent of *NbBSLs.* The efficiency of *NbBSLs* silencing was verified using qRT-PCR, t-test (*P* < 0.05). Hypersensitive response (HR) was observed, and images were captured 7 days after infiltration (*n* = 30)

**Fig. 2:**
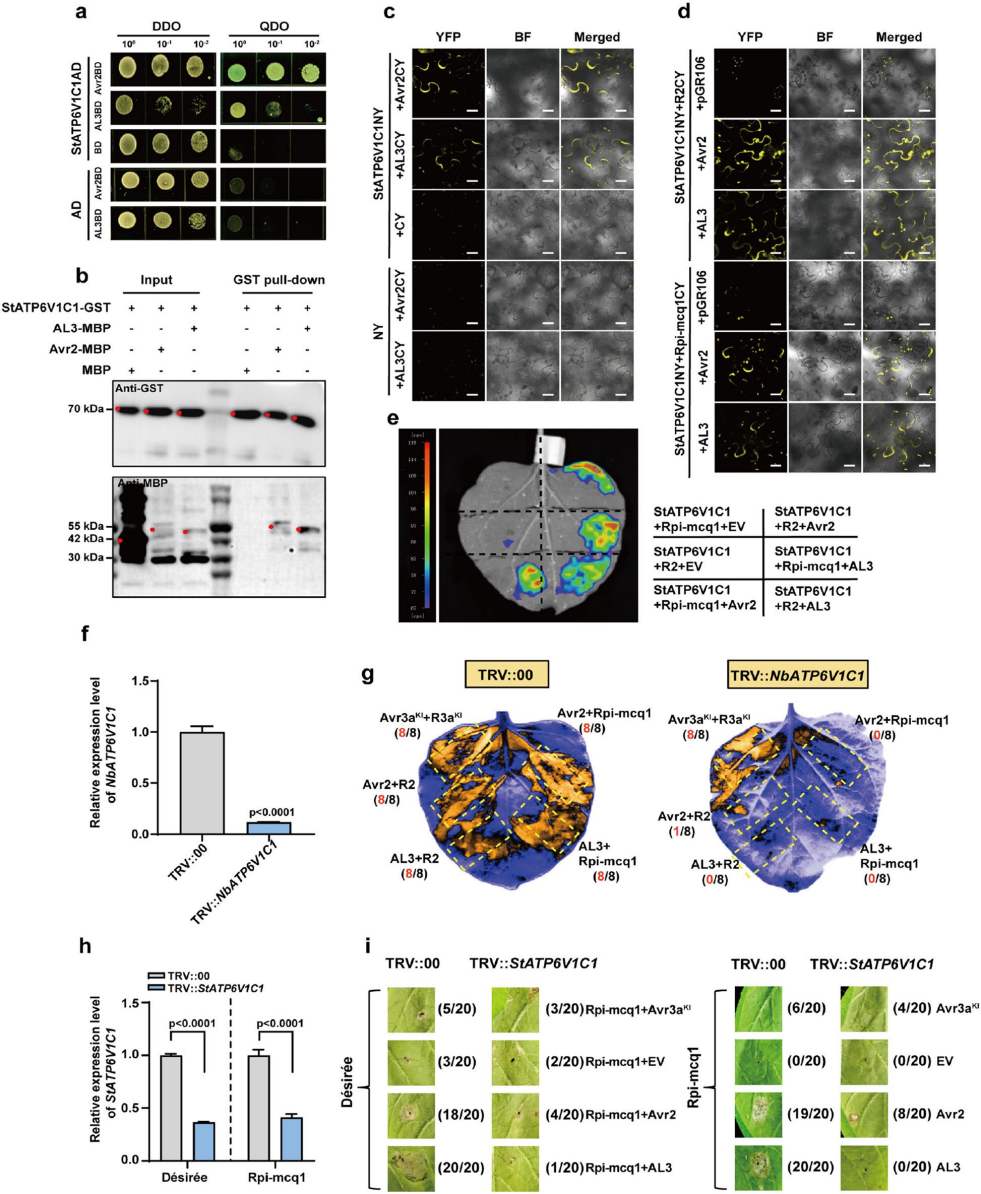
StATP6V1C1 is necessary for the recognitions between R2/Rpi-mcq1 and Avr2/AL3. **a**-**c**, Both Avr2 and AL3 interacted with StATP6V1C1, scale bars represent 20 µm. **d**-**e**, Both Avr2 and AL3 induced the interactions between StATP6V1C1 and the two R proteins (R2 and Rpi-mcq1), scale bars, 20 µm. The experimental groups included the empty vector (EV, pGR106), Avr2/AL3 (pGR106-Avr2/AL3), StATP6V1C1 (StATP6V1C1-cLUC), and R2/Rpi-mcq1 (R2/Rpi-mcq1-nLUC), *n* = 8, t-test (*P* < 0.05). **f**-**g**, Silencing of *NbATP6V1C1* attenuated the HR triggered by the recognition between Avr2/AL3 and Rpi-mcq1/R2. The efficiency of *NbATP6V1C1* silencing was verified by qRT-PCR, t-test (*P* < 0.05). R3a^KI^-Avr3a^KI^ served as a negative control. Graphs were generated 7 days after infiltration, *n* = 8. **h**-**i**, *StATP6V1C1* was required for Rpi-mcq1-Avr2/AL3 recognition. The efficiency of *StATP6V1C1* silencing was confirmed using qRT-PCR. Avr2, AL3, pGR106 (EV, negative control), and Avr3a^KI^ (negative control) were co-expressed with Rpi-mcq1 in ‘Désirée’, or expressed separately in transgenic potato plants harboring the *Rpi-mcq1* gene. Graphs were generated 7 days post-infiltration, with a sample size of 20

We performed an agroinfiltration transient assay on Nicotiana benthamiana leaves. In the presence of immunoreceptor R2, four effectors, PITG_22870 (Avr2), PexRD11, PITG_21949, and PITG_23008 (AL3) triggered HR. Two of them, Avr2 and AL3, triggered HR in the presence of immunoreceptor Rpi-mcq1 (Fig. 1a), which has been reproduced in Rpi-mcq1 transgenic *N. benthamiana* and potato leaves (Fig. S2a; Fig. 2i). In addition, PITG_07499 induced auto-necrosis, which is consistent with previously reported results (Saunders et al. 2012).

Avr2 and AL3 share a common structural composition, including a putative conserved signal peptide (SP), an RxLR-dEER motif, and a C-terminus (Fig. S1a), however, they exhibit significant differences in sequence and structure (Fig. S1a,b). Similar to the virulence function of Avr2 (Turnbull et al. 2019), transient overexpression of AL3 markedly suppressed potato immunity to late blight, as did the expression of three other members: PITG_21949, PITG_06077, and PITG_07499 (Fig. S2b,c). Both Avr2 and AL3 exhibit decentralized subcellular localization, including the nucleus, cytoplasm, and near the cell membrane (Fig. S2d), which provides the possibility that they may target multiple targets.

### AL3 is recognized by Rpi-mcq1 in a non-BSL-dependent manner

Previous studies have revealed that Avr2 is associated with BSL1 and mediates the interaction between BSL1 and R2 in planta, possibly by forming a ternary complex, which plays a key role in R2-mediated resistance (Saunders et al. 2012). Two other members of the BSL protein family, BSL2 and BSL3 (BSL2/3) play a role in Rpi-mcq1-Avr2 recognition (Turnbull et al. 2019; Wang et al. 2021). To explore whether StBSLs also play a role in the interactions between AL3 and R2/Rpi-mcq1, we first demonstrated the direct interaction between AL3 and the phosphatase domain of StBSL1 through Y2H (Fig. 1b), pull-down (Fig. 1c), and BiFC (Fig. 1d) assays. Similar to the Avr2-BSL1-R2 complex model, AL3 promotes the interaction between StBSL1 and both NLRs (R2 and Rpi-mcq1) in planta (Fig. 1e,f; Fig. S2e,f; Fig. S3a), suggesting that StBSL1 may play a conserved role in the recognition of Avr2 and AL3 by R2.

There is no direct interaction between R2/Rpi-mcq1 with Avr2/AL3, for their recognitions, intermediate factor is necessary (Fig. S3b,c). To investigate whether BSLs are required for R2/Rpi-mcq1-mediated perception of AL3, we conducted transient co-expression assays on TRV::*NbBSL1* (silencing efficiency of 79%) and TRV::*NbBSL2/3* (silencing efficiency of 73%) plants (Fig. 1g). R2-AL3/Avr2 recognition is dependent on NbBSL1/2/3, whereas Rpi-mcq1-Avr2 recognition relies on NbBSL2/3. Interestingly, silencing the NbBSLs had almost no effect on Rpi-mcq1-AL3 recognition (Fig. 1g,h,d). A recognition assay was also performed on potato plants, with silencing efficiencies of 77% and 62% for TRV::*StBSL1* and TRV::*StBSL2/3*, respectively (Fig. S3e). Consistent with the above results, StBSLs are required for the recognition of R2-Avr2/AL3 and Rpi-mcq1-Avr2, but not for the recognition of Rpi-mcq1-AL3 (Fig. 1h; Fig. S3f).

### StATP6V1C1 is required for Rpi-mcq1/R2-Avr2/AL3 recognition

To identify the intermediate protein that mediates Rpi-mcq1-AL3 recognition, we used a potato yeast two-hybrid cDNA system for screening. Subunit C of V-ATPase (ATP6V1C1) was selected as the candidate target of AL3 with the highest frequency. ATP6V1C1 is required for V-ATPase assembly and proton channel formation, and it is directly responsible for the binding and transmembrane transport of protons in plant cells (Zhou et al. 2018; Wang et al. 2023). The amino acid sequence of ATP6V1C1 is highly conserved across plant species (Fig. S4). Both Avr2-StATP6V1C1 and AL3-StATP6V1C1 interactions were validated by Y2H (Fig. 2a), pull-down (Fig. 2b), and BiFC (Fig. 2c) assays, suggesting that StATP6V1C1 is a potential common target for Avr2 and AL3. Similar to StBSLs, no direct interaction was observed between StATP6V1C1 and R2/Rpi-mcq1 (Fig. S5a), interestingly, both Avr2 and AL3 can activate the StATP6V1C1-R2/Rpi-mcq1 interactions (Fig. 2d,e; Fig. S5b,c).

To investigate the role of ATP6V1C1 in R2/Rpi-mcq1-Avr2/AL3 recognition, we silenced the *NbATP6V1C1* genes (with a silencing efficiency of 88%) and conducted co-expression assays (Fig. 2f). VIGS *NbATP6V1C1* did not diminish HR triggered by Avr3a^KI^-R3a^KI^ which is another potato and *P. infestans* R-Avr recognition pair. All four co-expressed groups (R2-Avr2, R2-AL3, Rpi-mcq1-Avr2, and Rpi-mcq1-AL3) elicited HR in the TRV::00 plants but failed to trigger HR in the TRV::*NbATP6V1C1* plants (Fig. 2g; Fig. S5d). Similarly, in potato plants, silencing *StATP6V1C1* diminished the four aforementioned recognition in both ‘Désirée’ and Rpi-mcq1 transgenic plants (Fig. 2h,i). Therefore, StATP6V1C1 is specifically involved in the Rpi-mcq1/R2-Avr2/AL3 recognition.

### StATP6V1C1 enhances potato resistance to late blight

**Fig. 3:**
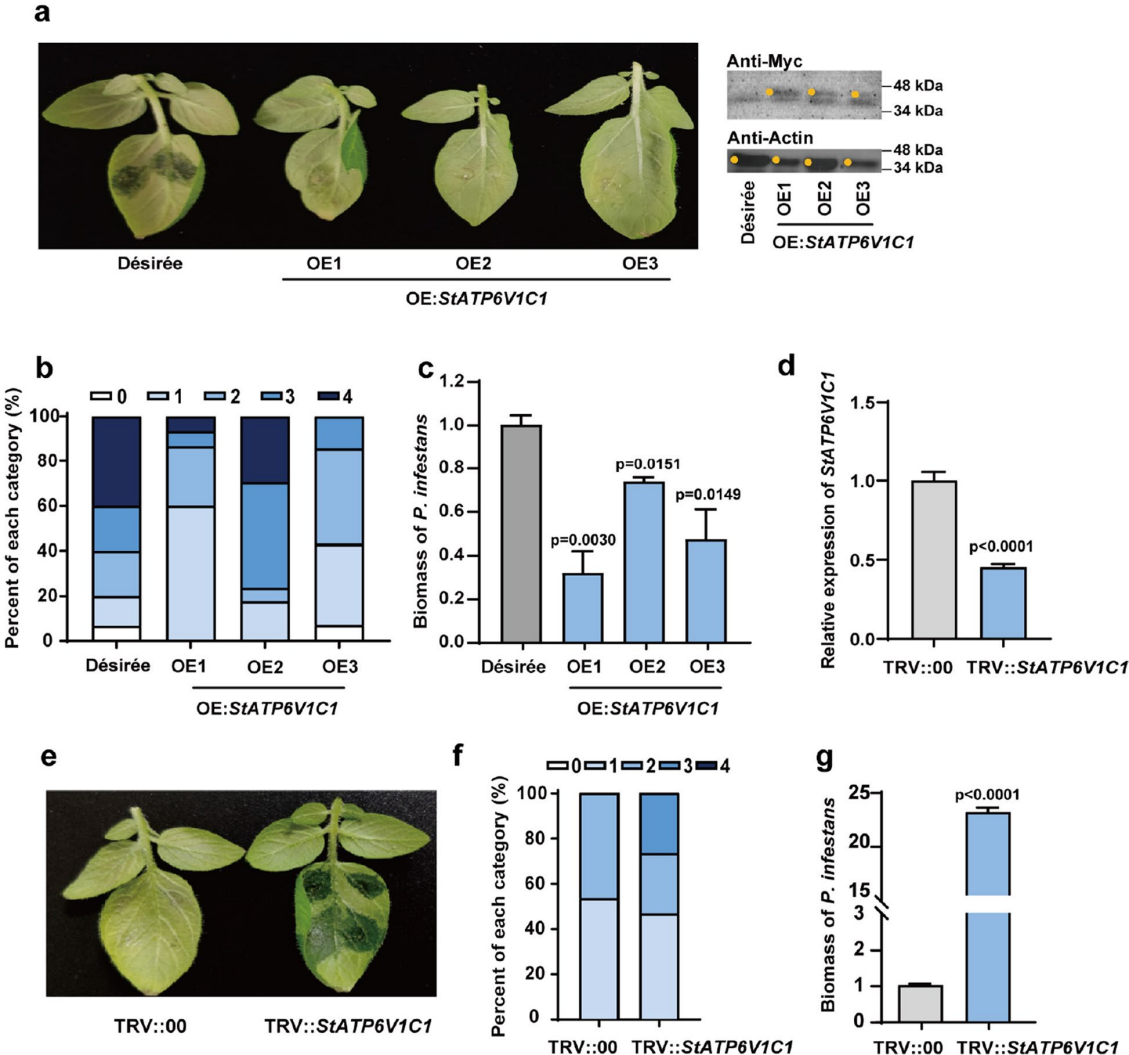
StATP6V1C1 positively regulates potato late blight resistance. **a**-**c**, Transgenic lines that overexpress *StATP6V1C1* demonstrated enhanced resistance to late blight. **d**-**g**, TRV::*StATP6V1C1* plants exhibited increased susceptibility to *P. infestans*. The efficiency of *StATP6V1C1* silencing was verified by qRT-PCR. The disease indices were assessed at 3 dpi, *n* = 30. The biomass of *P. infestans* was measured at 5 dpi, t-test (*P* < 0.05)

We investigated the role of StATP6V1C1 in resistance to potato late blight through over-expression and VIGS assays. The disease indices of the three overexpression transgenic lines (40%, 50%, and 41%, respectively) were significantly lower than that of the wild-type ‘Désirée’ (68%) at 72 hpi, and the *P. infestans *biomass of the three lines was also lower (26%, 68%, and 52%, respectively) than that of ‘Désirée’ at 5 dpi (Fig. 3a-c). Consistent with the disease resistance phenotype, *StATP6VC1* transgenic lines presented greater *StPR5* expression than did the wild-type at 2 dpi (Fig. S5e). The disease indices of the transient overexpression lines and the empty vector control were 41% and 70%, respectively, at 4 dpi (Fig. S5f,g). In addition, the disease index of the TRV::*StATP6V1C1* plants (45%) was greater than that of the TRV::00 plants (37%) at 60 hpi, and the *P. infestans* biomass of the TRV::*StATP6V1C1* plants was 22.7 times greater than that of the TRV::00 plants at 5 dpi (Fig. 3d-g). Overall, StATP6V1C1 positively regulates potato resistance to late blight.

### Avr2 and AL3 bidirectionally manipulate the V-ATPase activity and intracellular pH

**Fig. 4:**
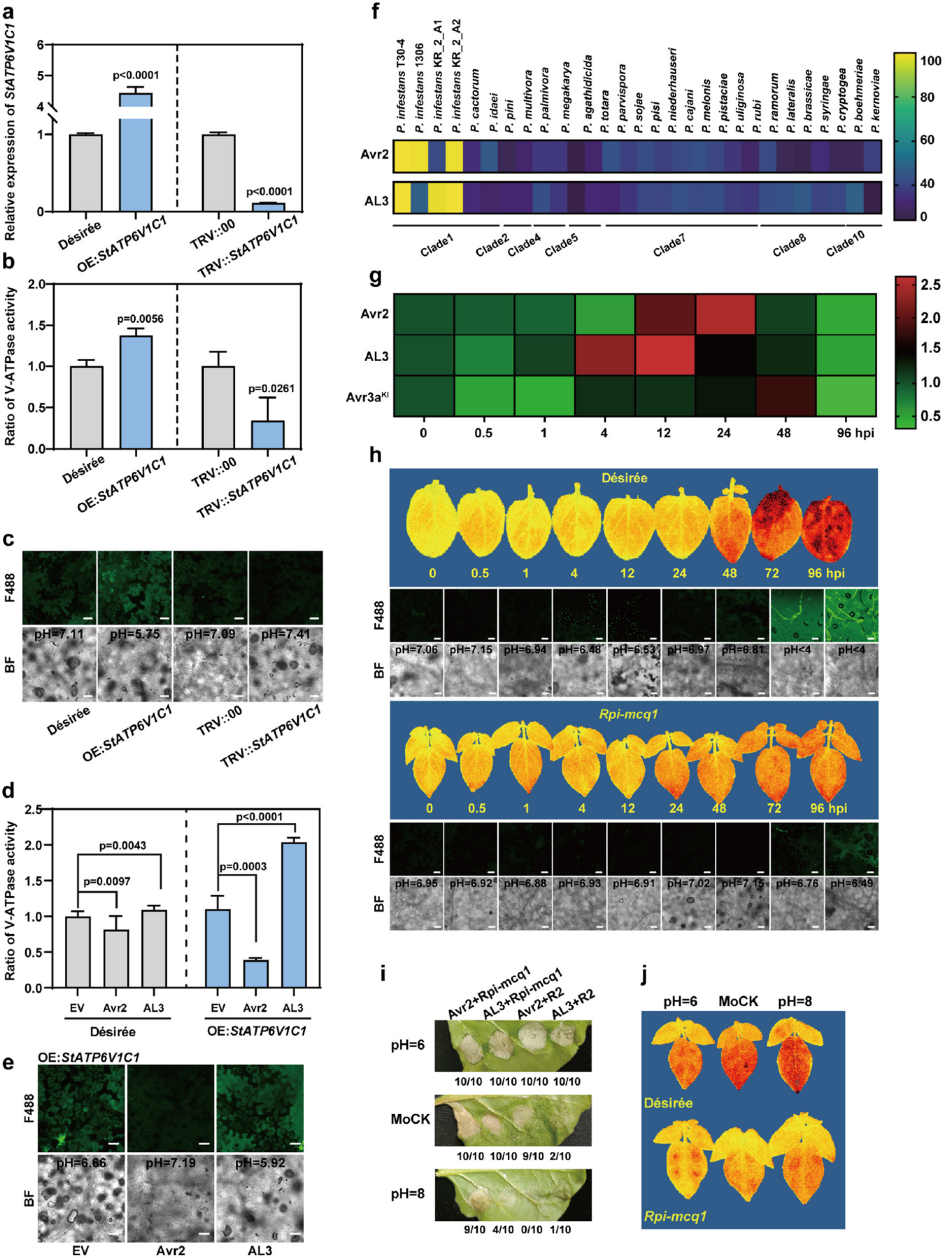
AL3 and Avr2 regulate V-ATPase activity and the intracellular pH level of the host. **a**-**c**, The expression level of *StATP6V1C1* regulates the activity of V-ATPase and intracellular pH. The relative expression levels of OE:*StATP6V1C1* and TRV::*StATP6V1C1* plants were confirmed using qRT-PCR. Data were collected from three experiments, each involving more than ten plants per group, t-test (*P* < 0.05). The intracellular pH was assessed in ten random microscopic fields from five leaves, with scale bars measuring 30 µm. **d**-**e**, Avr2 and AL3 bidirectionally manipulated plant V-ATPase activity and intracellular pH level. Data were derived from ten plants for each treatment and are presented as means, t-test (*P* < 0.05), scale bars, 30 µm. **f**, Avr2 and AL3 evolved specifically in *P. infestans*. The amino acid sequences of Avr2 and AL3 were compared for protein homology with the genomic sequences of 4 *P. infestans* strains and 24 other *Phytophthora* strains. The genome sequences were retrieved from the NCBI database (https://www.ncbi.nlm.nih.gov/genome/) and the *Phytophthora* Genome Sequencing Consortium download website (https://phyto-seq.cqls.oregonstate.edu). **g**, Sequential expression patterns of Avr2 and AL3 induced by *P. infestans*. Eight-week-old ‘Désirée’ plants were utilized for the inoculation of *P. infestans* EC1. The pathogen* β-tubulin* gene served as an internal reference, and the transcription levels were detected at 0, 0.5, 1, 4, 12, 24, 48, and 96 hpi. **h**, EC1-induced intracellular pH fluctuations in the leaves of ‘Désirée’and *Rpi-mcq1* transgenic plants. Scale bars represent 30 µm. **i**, Intracellular pH influenced R-Avr recognition. HR was observed, and images were captured five days after infiltration. **j**, Intracellular pH influenced potato resistance to late blight. Leaves were pre-infiltrated with gradient pH buffers one day before inoculation

ATP6V1C1 subunit plays a crucial role in regulating V-ATPase assembly and intracellular pH levels. Overexpression of *StATP6V1C1 *resulted in an increase in V-ATPase activity and a decrease in intracellular pH from 7.11 to 5.75. Conversely, in the TRV::*StATP6V1C1* plants, V-ATPase activity was reduced, leading to an increase in intracellular pH from 7.09 to 7.41 (Fig. 4a-c).

To determine whether Avr2/AL3 targets StATP6V1C1 to manipulate the host's intracellular pH level, we expressed Avr2/AL3 in both ‘Désirée’ and OE:*StATP6V1C1* plants. Although both Avr2 and AL3 interacted with StATP6V1C1, Avr2 inhibited V-ATPase activity and increased the intracellular pH of OE:*StATP6V1C1* plants from 6.66 to 7.19. In contrast, AL3 enhanced V-ATPase activity and decreased the intracellular pH of OE:*StATP6V1C1* plants from 6.66 to 5.92 (Fig. 4d,e). In order to further validation, we independently transiently expressed the pTA7001-Avr2 and pTA7001-AL3 constructs in *N. benthamiana* leaves. After DEX treatment for 12 h, the intracellular pH of the leaves expressing Avr2 increased from 6.51 to 7.09, while the intracellular pH of the leaves expressing AL3 decreased from 6.50 to 5.95 (Fig. S5h,i).

Hypothetical Avr2 and AL3 proteins were screened from the sequenced genomic DNA of four strains of *P. infestans* and 24 species of *Phytophthora*. Genome analysis of these *Phytophthora* species revealed that Avr2 and AL3 are conserved within the genomes of *P. infestans* (Fig. 4f). When the pathogen β-tubulin gene was used as an internal reference, the transcription level of *AL3* peaked after 12 hpi (hours post infection), whereas the expression level of *Avr2* peaked at 24 hpi (Fig. 4g). The intracellular pH of potato leave cells was assessed using BCECF-AM at various time points following infection with *P. infestans* EC1. Intracellular acidification occurs at 4 hpi, followed by intracellular alkalinization around 24 hpi (Fig. 4h). This suggests that *P. infestans *may evolve Avr2 and AL3 to sequentially regulate intracellular pH at different stages of the infection process.

Interestingly, in the leaves of Rpi-mcq1 transgenic lines, no similar pattern was observed. We also found that changes in intracellular pH may affect the R-Avr recognition and plant disease resistance. An acidic intracellular environment appears to be more conducive to R-Avr recognition, particularly in R2-related processes (observed 5 days after Agrobacterium infection) (Fig. 4i). In both ‘Désirée’ and Rpi-mcq1 plants, acidified leaves exhibited greater resistance to late blight compared to alkalized leaves (Fig. 4j). Meanwhile, a series of pH-dependent experimental results indicated that *P. infestans* infestation was more severe in alkalized* N. benthamiana* leaves (Fig. S6a-c).

### AL3 facilitates the assembly of StATP6V1C1 with StATP6V1G and StATP6V1E

**Fig. 5:**
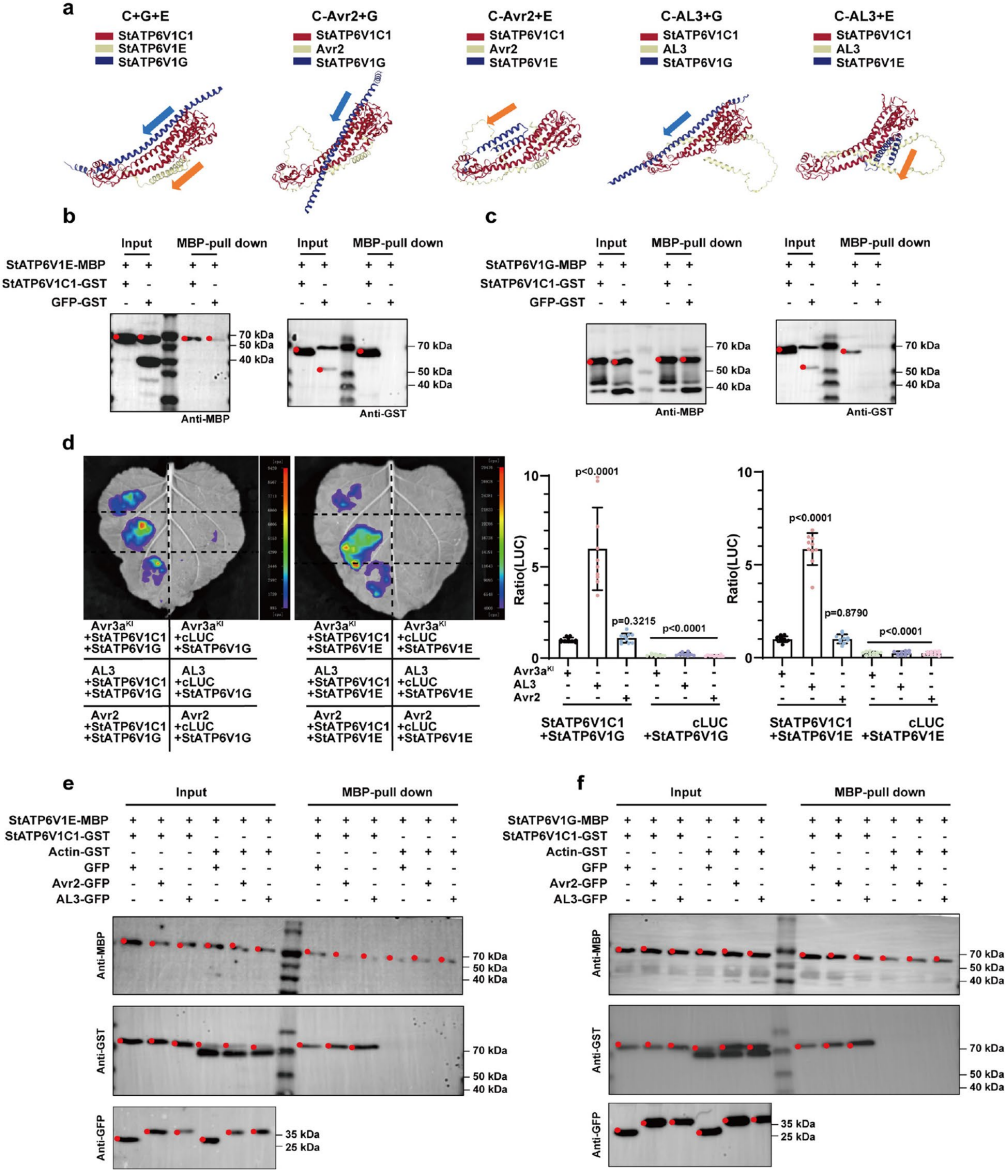
AL3 promotes the G-C-E assembly of V-ATPase. **a**, Molecular docking models of (StATP6V1C1-Avr2/AL3)-StATP6V1G/StATP6V1E. **b**, MBP pull-down assay of StATP6V1C1 and StATP6V1G. **c**, MBP pull-down assay of StATP6V1C1 and StATP6V1E. **d**-**f**, LCI and MBP pull-down assays demonstrated that AL3 can enhance the interaction between StATP6V1C1 and StATP6V1G/StATP6V1E. Avr3a^KI^/AL3/Avr2 (pGR106-*Avr3a*^*KI*^/*AL3*/*Avr2*), StATP6V1C1 (pCAMBIA1300-cLUC-*StATP6V1C1*), StATP6V1G/StATP6V1E (pCAMBIA1300-nLUC-*StATP6V1G/StATP6V1E*), *n* = 10, t-test (*P* < 0.05)

ATP6V1C1 is essential for the assembly of V-ATPase and the formation of proton channels (Zhou et al. 2018; Seidel et al. 2022; Wang et al. 2023). The StATP6V1G and StATP6V1E subunits are components of the peripheral stalk and are fundamental for correct V-ATPase assembly. We employed molecular docking technology to predict the potential effects of Avr2/AL3 binding on the assembly of the three subunits. When targeted by Avr2, StATP6V1C1 was predicted to interact with StATP6V1G and StATP6V1E in improper orientations (Fig. 5a). Based on direct interactions between StATP6V1C1 and StATP6V1G/StATP6V1E (Fig. 5b,c; Fig. S6d,e), LCI and pull-down assays revealed that the interactions of both C-G and C-E could be enhanced by AL3 (Fig. 5d-f; Fig. S6f), which is anticipated to facilitate the assembly of the V-ATPase complex.

### Avr2 inhibits the Ser261 phosphorylation of StATP6V1C1, which is catalyzed by StWNK10

**Fig. 6:**
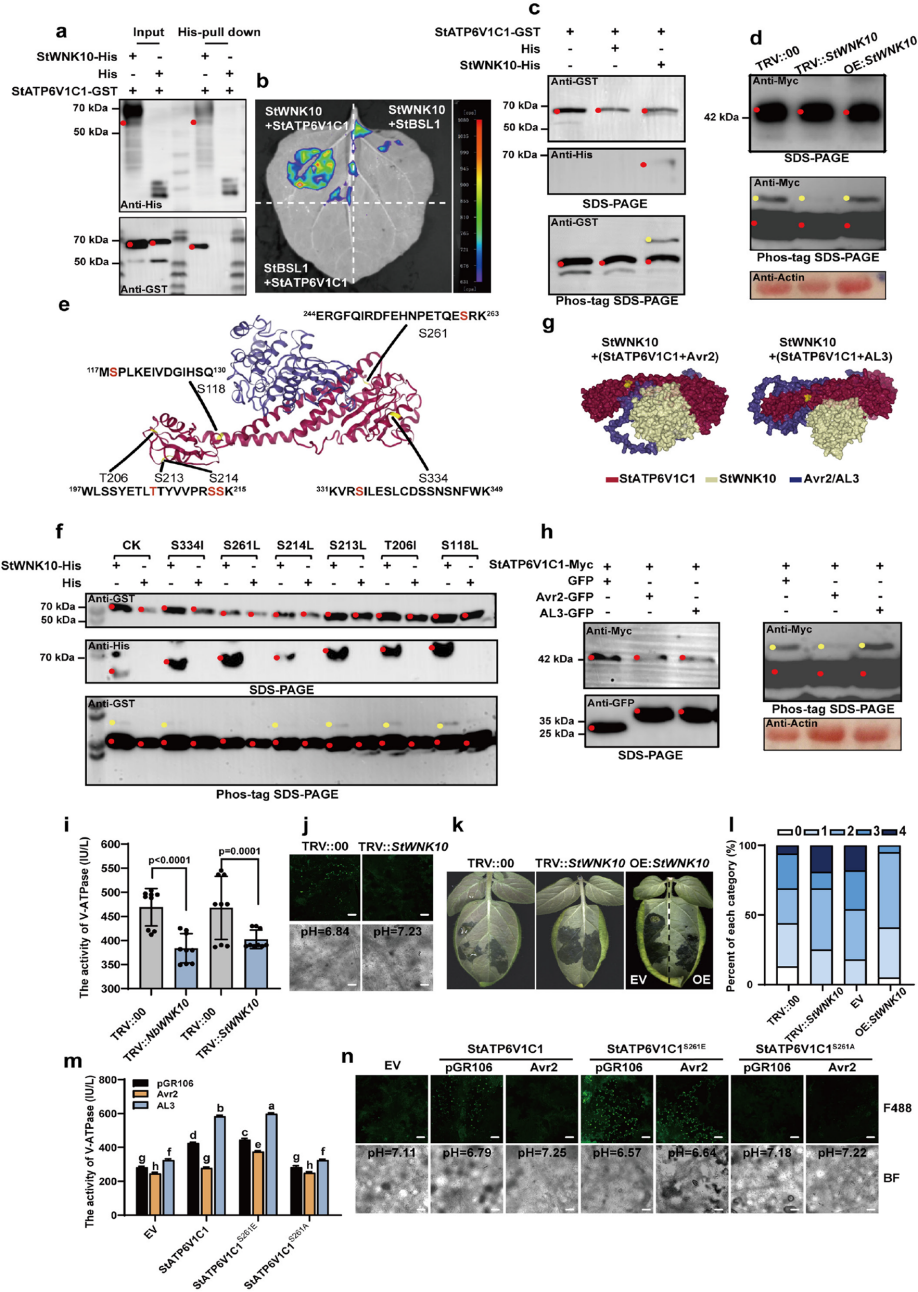
Avr2 prevents the S261 phosphorylation of StATP6V1C1 catalyzed by StWNK10. **a**-**b**, StWNK10 interacted with StATP6V1C1. **c**, In vitro phosphorylation assays demonstrated that StWNK10 directly phosphorylates StATP6V1C1. **d**, The silencing of *StWNK10* inhibited the phosphorylation of StATP6V1C1 in vivo. **e**, WNK kinase-specific phosphorylation site prediction for StATP6V1C1. The predicted phosphorylation sites are highlighted in yellow. **f**, StATP6V1C1^S261L^ mutant protein evades phosphorylation by StWNK10 in vitro. **g**, Avr2 was anticipated to directly inhibit the binding between StWNK10 and the StATP6V1C1^S261^ site due to steric hindrance. The S261 site is in yellow. **h**, Avr2 inhibited the phosphorylation of StATP6V1C1 in vivo. **i**, The silencing of *WNK10* resulted in a downregulation of V-ATPase activity, *n* = 9, t-test (*P* < 0.05), scale bars = 30 µm. **j**, The silencing of *StWNK10* induced intracellular alkalization in potato leaves. **k**-**l**, *StWNK10* positive regulated potato late blight resistance. Representative leaves were photographed three days after *P. infestans* infection. **m**, The phosphorylation of StATP6V1C1^S261^ site is crucial for V-ATPase activity. **n**, The StATP6V1C1^S261^ site is crucial for intracellular acidification and the regulation of Avr2, scale bars, 30 µm

The mechanism underlying the assembly and activation of the V-ATPase remains unclear. Previous reports indicate that Arabidopsis AtWNK8 binds to and phosphorylates AtATP6V1C1 and other V-ATPase subunits (Hong-Hermesdorf et al. 2006). Based on phylogenetic tree analysis, we cloned the full-length StWNK10 gene from the ‘Désirée’ (Fig. S6g). The interaction between StWNK10 and StATP6V1C1 was confirmed through pull-down and LCI experiments (Fig. 6a,b; Fig. S7a). An in vitro phosphorylation assay demonstrated that StWNK10 functions as a phosphokinase for StATP6V1C1 (Fig. 6c). Silencing StWNK10 in OE:*StATP6V1C1*-Myc potato plants resulted in a weakened phosphorylated band of StATP6V1C1 (indicated by the yellow dot) (Fig. 6d). Previous studies identified six candidate WNK-specific phosphorylation sites on AtATP6V1C1 through matrix-assisted laser desorption ionization time-of-flight mass spectrometry (MALDI-ToF–MS) analysis. The comparison of amino acid sequences identified the S118, T206, S213, S214, S261, and S334 residues as potential phosphorylation sites of StATP6V1C1 (Fig. 6e) (Hong-Hermesdorf et al. 2006). Next, we generated six point mutations by substituting serine or threonine with isoleucine or leucine at each previously mentioned site. Western blot analysis confirmed the expression of these mutant proteins (Fig. S7b). In vitro, kinase assays further confirmed that StATP6V1C1^S261^ is the phosphorylation site for StWNK10 (Fig. 6f). In the molecular docking model, the binding of Avr2, compared to AL3, inhibited the S261 phosphorylation of the StATP6V1C1 (Fig. 6g). It can partially explain why Avr2, rather than AL3, suppressed the phosphorylation of StATP6V1C1 in vivo (Fig. 6h).

To investigate the role of StWNK10 in regulating V-ATPase activity and plant intracellular pH, the *StWNK10* gene was successfully silenced in *N. benthamiana* and potato plants, achieving silencing efficiencies of 72% and 68%, respectively. V-ATPase activity was reduced by 18% in TRV::*NbWNK10* plants and by 14% in TRV::*StWNK10* plants (Fig. 6i). Consequently, the intracellular pH of TRV::*StWNK10* plants was higher compared to that of TRV::00 plants (Fig. 6j). The disease index and *P. infestans *biomass in TRV::*StWNK10* plants (disease index of 56.25%) were greater than those in TRV::00 plants (disease index of 45.31%) (Fig. 6k,l; Fig. S7c). Results from the half-leaf disease experiment indicated that the overexpression of *StWNK10* enhanced potato resistance to late blight, resulting in a lower disease index (39.77% compared to the EV control group's index of 61.36%) and a reduced biomass of *P. infestans* (51.43%) (Fig. 6k,l; Fig. S7d).

To further investigate the role of S261 phosphorylation in regulating cellular pH in plants, we introduced the S261E mutation to simulate the phosphorylated state and the S261A mutation to represent the dephosphorylated state. Compared to the control group, the overexpression of StATP6V1C1^S261E^ enhanced V-ATPase activity and promoted intracellular acidification in* N. benthamiana*. In contrast, the overexpression of StATP6V1C1^S261A^ appeared to have no effect on V-ATPase activity or intracellular pH (Fig. 6m,n). When co-expressed with StATP6V1C1, Avr2 significantly inhibited V-ATPase activity and acidified the* N. benthamiana* cellular environment. However, when StATP6V1C1 was persistently phosphorylated, the Avr2-mediated inhibitory effect was diminished, resulting in cells maintaining a slightly acidic cellular environment (Fig. 6m,n). Moreover, we transiently expressed StATP6V1C1^S261^, StATP6V1C1^S261E^, and StATP6V1C1^S261A^ in potato leaves and inoculated them with EC1 on detached leaves. StATP6V1C1^S261A^ (disease index 85%) had little effect on potato late blight resistance compared to the control group (disease index 88.75%), while both StATP6V1C1^S261^ (disease index 56.25%) and StATP6V1C1^S261E^ (disease index 47.5%) significantly enhanced resistance (Fig. S7e-g). These results collectively indicate that the phosphorylation of StATP6V1C1^S261^ may play a crucial role in mediating the Avr2-dependent inhibition of V-ATPase activity and in the resistance of potatoes to late blight.

### pH shaking facilitates the targeting of Avr2/AL3 to the susceptibility factors StBSLs

Previous research has indicated that Avr2 activates Brassinosteroid (BR) signaling by stimulating BSL1 activity, thereby tipping the balance between growth and immunity, which promotes potato late blight disease (Turnbull et al. 2017). We also observed that the compound leaves of OE:*StATP6V1C1* plants are more susceptible to abnormalities, including missing and adhesions. Several BR-inducible genes were upregulated in the *StATP6V1C1* overexpression lines compared to the wild type, particularly. This gene is known to facilitate the antagonism between BR and immune responses, and it plays a crucial role in Avr2 activity (Fig. S8a-f) (Turnbull et al. 2017). This suggests a correlation between the expression of *StATP6V1C1* and BR signaling.

**Fig. 7:**
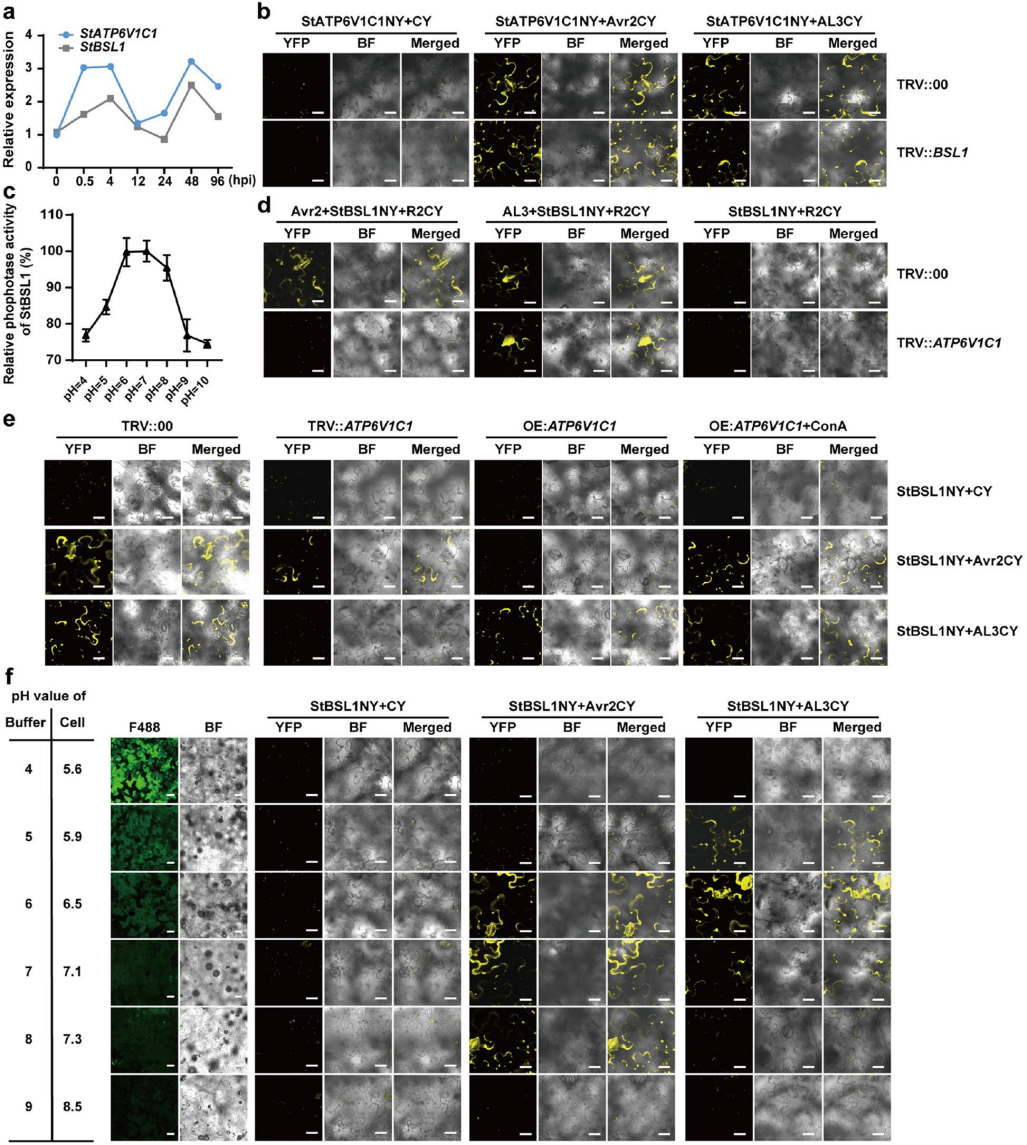
AL3 and Avr2 induce pH fluctuations and promote targeting the susceptibility factor StBSL1. **a**, Pathogen-induced expression patterns of *StATP6V1C1* and *StBSL1*. **b**, Silencing of *BSL1* did not affect the interactions between StATP6V1C1 and Avr2/AL3, scale bars, 20 µm. **c**, The enzyme activity of StBSL1 was assessed across a pH range of 4 to 10, *n* = 3. **d**, The interaction of StBSL1-R2 (induced by Avr2) is dependent on the expression of *ATP6V1C1*, scale bars, 20 µm. **e**, The expression level of *ATP6V1C1* influences the interaction between StBSL1 and Avr2/AL3, scale bars, 20 µm. **f**, Intracellular pH influenced the interactions of StBSL1-Avr2/AL3. Gradient pH buffers (10 mM MgCl_2_, pH range of 4–10) were infiltrated into *N. benthamiana* leaves to adjust the cellular pH from 5.64 to 8.51, scale bars, 20 µm

Both StATP6V1C1 and StBSL1 are involved in the recognition of R2-Avr2/AL3, and their expression patterns exhibited a high correlation coefficient of 0.86 (Fig. 7a). The silencing of BSL1 did not affect the interactions between StATP6V1C1 and Avr2/AL3 (Fig. 7b). BSL1 is an alkaline phosphatase whose activity is precisely regulated by pH levels. The interaction between the putative phosphatase domain of BSL1 and Avr2 in planta has been documented (Mora-García et al. 2004; Saunders et al. 2012). The enzyme activity of StBSL1 was investigated using the chromogenic substrate p-nitrophenylphosphate (pNPP) method, with an optimal pH of 7 (Fig. 7c). Interestingly, the overexpression of *ATP6V1C1* disrupted the interaction between StBSL1 and Avr2. This interaction can be restored by the V-ATPase activity inhibitor Concanamycin A (Con A). Conversely, silencing of ATP6V1C1 also disrupted the interaction between StBSL1 and AL3. Additionally, the Avr2-induced interaction between R2 and StBSL1 was absent in the TRV::*ATP6V1C1* plants (Fig. 7d,e).

To investigate whether StATP6V1C1 modulates the interactions between StBSL1 and Avr2/AL3 by regulating cellular pH levels, we infiltrated a series of standard pH buffers into the leaves of *N. benthamiana *and established a cellular pH gradient ranging from 5.6 to 8.5. The interaction between StBSL1 and Avr2 was maintained within a pH range of 6.5 to 7.3, implying that it is more stable when the intracellular environment was alkalized (Fig. 7f). In contrast, the interaction between StBSL1 and AL3 interaction was sustained within the range of 5.9 to 7.1, indicating that it is more stable in acidic intracellular environments (Fig. 7f). Overall, in the absence of downstream R proteins, Avr2 and AL3 regulated the intracellular pH levels of host plants in opposing directions, thereby creating favorable conditions for the targeting of StBSL1.

## Discussion

**Fig. 8:**
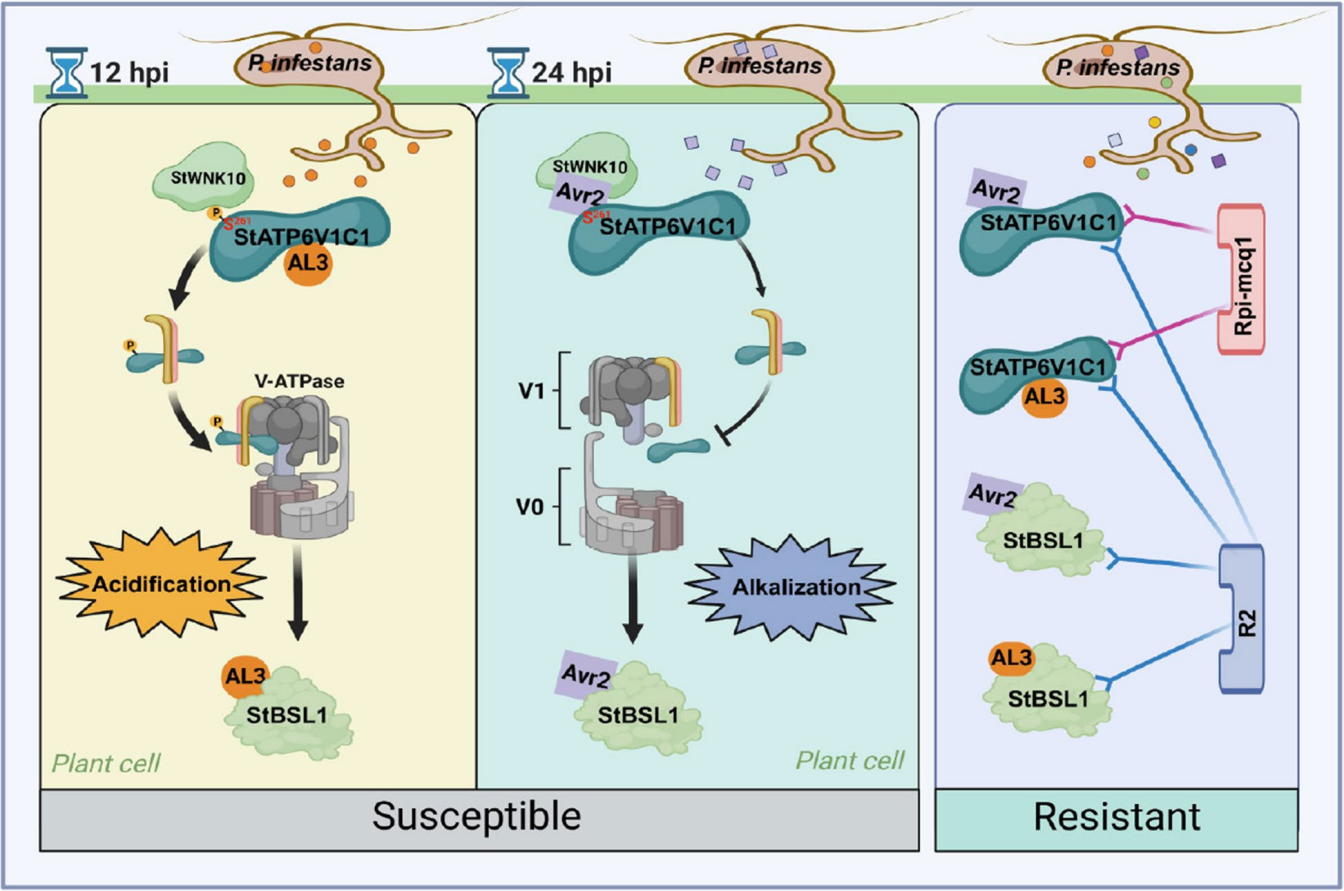
Models illustrating a bidirectional regulatory mechanism and immune function of VHA-C-mediated intracellular pH fluctuations. Two *Phytophthora infestans* RxLR effectors, AL3 (PITG_23008) and Avr2, are expressed sequentially and interact with the host protein VHA-C (StATP6V1C1) to bidirectionally regulate the assembly of V-ATPase and intracellular pH level. In the absence of two NLR proteins, this pH fluctuation facilitated the interactions between the two effectors and the downstream susceptibility factor StBSL1. Two NLRs, R2 and Rpi-mcq1, can monitor both the upstream StATP6V1C1 and downstream StBSL1 to recognize the effectors Avr2 and AL3, thereby reducing the risk of late blight in potatoes. The black bold arrow represents “promotion”, the black “T” arrow represents “inhibition”, red and blue “Y” arrows represent interactions

pH fluctuations play a key role in the battle between plants and pathogens, however, most studies have focused on P-ATPase-mediated changes in the pH levels of the apoplast (Batoko et al. 1998; Zhou et al. 2000; Seo et al. 2023). In this study, we focused on the *P. infestans*-plant interaction model, revealing a sequential and bidirectional regulatory mechanism that involves StATP6V1C1-mediated pH fluctuations in the intracellular environment. Additionally, we assessed its role in both the ETS and ETI processes (Fig. 8).

As an intracellular pH regulator, V-ATPase is a large multisubunit enzyme that contains several potential binding sites for Avr and R proteins (Hu et al. 2023; Wang et al. 2023; Pu et al. 2024). Previous studies have demonstrated that the Salmonella effector SopF disrupts the infection-induced association between V-ATPase and ATG16L1. This disruption promotes replication and ADP-ribosylates Gln124 of ATP6V0C in the V-ATPase, thereby inhibiting bacterial autophagy in mammals (Xu et al. 2019). In plant-pathogen interaction, BSMV replicase γα disrupts the interaction between VHA-B2 and VHA-E, thereby suppressing the V-ATPase activity and facilitating viral infection (Yang et al. 2022). NbATP6V0C is involved in the destabilization of NbFAD4 and negatively regulates TuMV infection (Fang et al. 2024). Our research is the first to reveal that oomycete effectors target the C subunit of VHA to promote pathogenesis. ATP6V1C1 has previously been demonstrated to regulate the reversible binding of the V1 and V0 domains in yeast and animals (Vasanthakumar et al. 2020; Seidel et al. 2022; Wang et al. 2023). In this study, we investigated the direct interaction between Avr2/AL3 and StATP6V1C1 through both in vitro and in vivo experiments (Fig. 2a-c). Any defect in the V1 subcomplex subunits may impact the binding of the V1-V0 sectors (Forgac et al. 2007; Vasanthakumar et al. 2020; Wang et al. 2023). Our findings indicate that AL3 promoted the assembly of StATP6V1C1 with the G and E subunits of the V1 domain (Fig. 5), increasing V-ATPase activity and results in intracellular acidification (Fig. 4d,e). AtWNK8 positively regulates V-ATPase by phosphorylating AtATP6V1C1 (Hong-Hermesdorf et al. 2006). Here, we reveal that StWNK10, the homologous protein of AtWNK8, phosphorylates StATP6V1C1 at the S261 site, thereby increasing V-ATPase activity and enhancing potato late blight resistance (Fig. 6; Fig. S6). Avr2 inhibited the phosphorylation of StATP6V1C1 and the activity of V-ATPase, resulting in intracellular alkalinization (Fig. 6g,h) (Hong-Hermesdorf et al. 2006). Thus, we demonstrated that AL3 and Avr2 bidirectionally regulate V-ATPase activity by modulating the assembly and phosphorylation of the complex.

During the arms race between plants and pathogens, pathogens have developed various mechanisms, including pH manipulation, to evade the host's immune system (Felix et al. 1999; Yamaguchi et al. 2006; Zipfel et al. 2006; Li et al. 2022; Liu et al. 2022). Both Avr2 and AL3 are important virulence factors unique to *P. infestans* (Fig. 3f; Fig. S2b,c). In the early stages of *P. infestans* infection, *AL3* exhibited the highest expression level at 12 hpi (Fig. 4g). This increase in *AL3* expression enhanced V-ATPase activity and lowered the intracellular pH from 6.66 to 5.92 (Fig. 4d,e). The expression level of *Avr2* subsequently peaked at 24 hpi (Fig. 4g). Avr2 inhibited V-ATPase enzymatic activity and increased the intracellular pH from 6.66 to 7.19 (Fig. 4d,e). The differences in the expression time windows of *Avr2* and *AL3* allow for the bidirectional regulation model of V-ATPase activity and intracellular pH.

In the absence of two NLR proteins, this effector-triggered pH fluctuation facilitated interactions between the two effectors and the downstream susceptibility factor StBSL1. We proved that a relatively acidic intracellular environment is more conducive to the AL3-StBSL1 interaction, while a slightly alkaline environment is more favorable for the Avr2-StBSL1 interaction (Fig. 7f). Thus, lacking immune receptors, *P. infestans* hijacks V-ATPase-regulated intracellular pH by secreting different effectors to promote disease establishment at different stages of infection. In the wild type, intracellular acidification induced by *P. infestans* occurs at 4 hpi, followed by intracellular alkalinization at 24 hpi (Fig. 4h). This suggests that *P. infestans* may evolve Avr2 and AL3 to sequentially regulate intracellular pH at various stages of the infection process. This mechanism does not seem to be present in Rpi-mcq1 transgenic lines, which may contribute to the associated resistance (Fig. 4h).

BSL1 is guarded by two immune receptors, R2 and Rpi-mcq1 (Aguilera-Galvez et al. 2019; Wang et al. 2021). Here, we further revealed that two NLRs also guard StATP6V1C1 to recognize Avr2 and AL3 (Fig. 2). In the silencing plants of ATP6V1C1, all of the four kinds R-Avr recognitions disappeared, while only R2-Avr2/AL3 recognitions were inhibited in TRV::NbBSL1 plants. Interestingly, the interactions between StBSL1-Avr2/AL3 and R2-StBSL1 (induced by Avr2) were significantly influenced by the expression level of ATP6V1C1 (Fig. 7d,e). This evidence indicates that StATP6V1C1 is upstream of StBSL1 in the recognition between R2 and Avr2/AL3. Additionally, the brassinosteroid-responsive bHLH transcription factor StCHL1 shows higher expression level in OE:*StATP6V1C1* plants (Fig. S8a-f). And other subunits of V-ATPase were found to maintenance of cellular pH and to be linked BR homeostasis to cause abnormal growth in Arabidopsis (Jiang et al. 2023). This suggests a correlation between the *StATP6V1C1*-OE caused leaf abnormalities and BR signaling.

We found that a slightly acidic intracellular environment is more conducive to several R-Avr recognitions, particularly the R2-Avr2 recognition. This finding was further confirmed in the alkaline intracellular environment of TRV::*ATP6V1C1* plants (Figs. 4i and 7d). In addition to R-Avr recognition, we also discovered that a slightly acidic intracellular environment is less favorable for the infection and colonization of *P. infestans* (Fig. 4j; Fig. S6a-c). This finding aligns with previous reports on other plant-pathogen interaction systems (Mathieu et al. 1996; He et al. 1998; Lapous et al. 1998; Fang et al. 2024). Both StATP6V1C1 and StWNK10 were found to acidify the intracellular environment and enhance potato resistance to late blight (Fig. 3; Fig. 6k-l; Fig. S5f-g; Fig. 6f). This study offers a novel theoretical basis for improving plant resistance through the regulation of V-ATPase activity and intracellular pH levels.

In summary, we revealed the cunning bidirectional modulation of *P. infestans* for virulence and proposed that plant immune receptors have evolved a more efficient countermeasure: monitoring both upstream and downstream of decoys (Fig. 8). Our research highlights the considerable potential applications of V-ATPase-mediated pH regulation in the prevention and control of plant diseases.

## Materials and methods

### Strains and plants

The Escherichia coli strains DH5α and BL21 were cultured on Luria–Bertani (LB) media at 37℃. The Agrobacterium tumefaciens strains GV3101 and AGL1 were cultured on LB media supplemented with 50 μg/mL rifampicin at 28℃. The *P. infestans* strains EC1 and 88,069 were cultured on Rye A and Rye B media at 18℃. Plants were cultivated under a 16-h/8-h night cycle at 22℃ in a climate chamber.

### Inoculation and disease assessment

EC1 was cultivated on Rye B agar media supplemented with 0.2 μg/mL β-sitosterol at 18℃ for 2 weeks. The mycelium and conidia were gently scraped into sterile water. The suspension was then incubated on ice for 2 h to facilitate the release of zoospores, with the concentration adjusted to 5 × 10^4^ zoospores per mL. Detached leaves were inoculated with 10 µL droplets on each side and kept in weak light at 21℃ (Wang et al. 2019).

Disease indices were measured at 3–5 dpi and scored as follows: 0, no visible infection; 1, < 25% infection; 2, 26–50% infection; 3, 51–75% infection; 4, 76–100% infection. The disease index = (number of diseased leaves × disease grade index)/(the total number of investigated leaves × 4) ${\textstyle\sum_0^4}xiyi/\left(\mathrm x\;\max\;\sum\;yi\right)$× 100% . The development of *P. infestans* was quantified by normalizing the *P. infestans* *PiO8* element values with the potato *StEF1α* gene values.

### RNA extraction and quantitative qRT-PCR

Total RNA was extracted from plant samples using the Plant RNA Kit (Omega Bio-tek, USA). First-strand cDNA was synthesized using the HiFiScript gDNA Removal RT MasterMix Kit (CWBIO, Beijing, China). Quantitative real-time PCR (qRT-PCR) was performed via an Archimed Analyzer (Rocgene, China) with the Ultra SYBR mixture (CWBIO). The StEF1α (LOC1026-00107) was used as an internal control. Gene expression levels were analyzed via the 2^−ΔΔCT^ method. The primers used for qRT-PCR are listed in Additional file 1.

### Protein extraction and pull-down assay

The full-length cDNAs of StATP6V1C1, StATP6V1C1^S334I^, StATP6V1C1^S261L^, StATP6V1C1^S214L^, StATP6V1C1^S213L^, StATP6V1C1^T206I^, StATP6V1C1^S118L^, GFP, and StActin were cloned and inserted into pGEX-4T-1 for the in vitro expression of recombinant proteins with an N-terminal GST tag. The cDNAs of Avr2, AL3, StATP6V1G, and StATP6V1E were cloned and inserted into pMAL-c2X for the in vitro expression of recombinant proteins proteins with an N-terminal MBP tag. The full-length cDNA of StWNK10 was cloned and inserted into pET-28a (+) for the in vitro expression of a recombinant protein with an N-terminal 6 × His tag. All the constructs were separately transformed into Escherichia coli BL21 (DE3) cells. The recombinant proteins were induced with 0.3 to 1 mM IPTG and harvested through ultrasonication and centrifugation.

The C-terminal domains of Avr2 and AL3 were fused with GFP, and the recombinant proteins were inserted into pCXSN-HA. Additionally, the full-length cDNA of *StATP6V1C1* was inserted into pCXSN-Myc. Protein extractions were performed using a Plant Protein Extraction Kit (CWBIO).

The crude proteins were separately mixed and incubated for more than 6 h in an ice bath. Pull-down assays were subsequently performed with either the Mag-Beads GST Fusion Protein Purification Kit (Sangon Biotech, Shanghai, China), the Dextrin Beads 6FF (Smart-Lifesciences, Changzhou, China) or the BeaverBeads™ His-tag Protein Purification (Beaver, Suzhou, China). The pulled-down proteins were separated by SDS-PAGE and detected by immunoblotting with an anti-GST (Proteintech), anti-His (CWBIO) or anti-MBP antibody (Proteintech).

### Yeast library screening and yeast two-hybrid (Y2H)

The yeast receptive state was prepared following the instructions outlined in the Matchmaker™ Gold Yeast Two-Hybrid System User Manual (Clontech, USA). The decoy protein was confirmed to be non-toxic and not self-activating. Yeast library screening was conducted according to the Clontech method. Positive clones were extracted using the TIANprep Yeast Plasmid DNA Kit and transformed into Escherichia coli DH5α for further culture expansion. Open reading frames (ORFs) were predicted and cloned. We used the Y2H method to confirm the identified protein interactions. The candidate genes were ligated into the prey vectors pGT7AD and pGBKT7. Transformants were identified via selection on media lacking Leu and Trp (DDO). Interactions were detected on media supplemented with X-α-gal, which lacks Leu, Trp, His and Ura (QDO).

### Bimolecular fluorescence complementation (BiFC)

The candidate genes were cloned and inserted into the pSPYNE and pSPYCE vectors (Walter et al. 2004). The constructs were transformed into GV3101. Five-week-old *N. benthamiana* leaves were agroinfiltrated, and images were captured via laser confocal fluorescence microscopy (Leica SP8, Germany).

### Luciferase complementation imaging (LCI) assay

pCAMBIA1300-nLUC and pCAMBIA1300-cLUC were used for the LCI assay (Chen et al. 2008). The constructs were transformed into GV3101. Five-week-old *N. benthamiana* leaves were agroinfiltrated. D-fluorescein potassium salt (1 mM) was sprayed onto the leaves, and the plants were incubated in the dark for 5 min. LUC images were captured via NightShade LB 985 (Berthold Technologies, Bad Wildbad, Germany).

### Virus-induced gene silencing (VIGS)

VIGS was conducted on plants following a previously described method (Liu et al. 2020). GV3101 carrying pTRV1 (RNA1) and various pTV00 (RNA2) plasmids were vacuum infiltrated into 1-week-old *N. benthamiana* and 2-week-old potato. VIGS efficiency was evaluated via qRT-PCR after 4 weeks.

### Agrobacterium-mediated transient and stable transgene expression

The transformed Agrobacterium GV3101 was diluted to OD_600_ = 0.5 ± 0.02 with diluent buffer (10 mM MgCl_2_, 150 mM acetosyringone, and 10 mM MES, pH = 5.7). After infiltration, the plants were incubated at 22℃ under dark and moist conditions for 12 h and then placed under normal conditions (16 h day/8 h night) for 2–3 days. We transformed the vector into potato for stable expression experiments using Agrobacterium-mediated stem segment transfor mations (Wang et al. 2019). Western blotting was performed to select the positive transgenic lines.

### Plant V-ATPase activity

The activity of the plant V-type proton pump enzyme was determined via the Plant V-ATPases ELISA Kit (Shanghai Fantai Biotechnology Co., Ltd. Shanghai, China).

### Cytosolic pH measurement

The 2’,7’-Bis-(2-carboxyethyl)−5-(and-6)-carboxyfluorescein, acetoxymethyl ester (BCECF- AM) fluorescence was detected via a confocal laser-scanning microscope (Halcrow et al. 2019; Zhao et al. 2022). The cytosolic pH was determined on the basis of the pH-dependent ratio of the emission intensity when the dye was excited at 488 nm (pH-dependent) to the emission intensity when it was excited at 448 nm (non-pH-dependent). The fluorescence signal intensity was calculated via ImageJ software. BCECF-AM was added to a final concentration of 10 μM in a series of pH buffer (10 MgCl_2_), which were used to treat the plant cells. The fluorescent signals were quantified and statistically analyzed. The standard curves were provided in Fig. S9.

### In vitro phosphorylation assay

StWNK10 kinase was incubated with the substrate StATP6V1C1 or with proteins modified by point mutations, for 60 min at 30℃ in a kinase assay buffer (50 mM Tris–HCl, pH = 7.5; 1 mM dithiothreitol; 10 mM MnCl_2_; 1 mM CaCl_2_; and 10 µM ATP). The reactions were stopped by adding 1 volume of 2 × SDS sample buffer (2% SDS, 80 mM Tris–HCl, 10% glycerol, 0.01% bromophenol blue, 0.1 M DTT, and 10% β-mercaptoethanol) and incubating at 98℃ for 5 min. Phosphorylation of StATP6V1C1 was verified via Phos-tag™ SDS-PAGE assay.

### Phosphatase activity assays

To immunoprecipitate His-BSL1, protein extracts were incubated with His-Trap beads (Beaver, Suzhou, China) for 1 h at 4℃. Beads were washed in 100 µl of p-nitrophenyl phosphate, disodium salt (PNPP) assay buffer (50 mM Tris–HCl, pH = 7.0, 0.1 mM CaCl_2_) after being washed twice with washing buffer (20 mM phosphate buffer, 500 mM NaCl, 60 mM imidazole, pH = 7.4). The beads were then resuspended in 25 µl of PNPP assay buffer supplemented with 2.8 mM MnCl_2_ and preincubated for 15 min at 32℃ before adding 30 µl of 5 mg/mL PNPP substrate (Sangon Biotech, China). Measurements were performed at 405 nm using a microplate reader (SpectraMax ID 5).

### Simulation and data processing

Protein models for StATP6V1C1, StATP6V1G, StATP6V1E, StWNK10, Avr2, and AL3 were proposed via SWISS-MODEL (www.ExPASy.org/resources/swiss-model) (Benkert et al. 2011; Waterhouse et al. 2018). The docking simulation was performed via GRAMM (accessed via https://gramm.compbio.ku.edu/) (Tovchigrechko et al. 2006). The StATP6V1C1 protein was further screened for the presence of a WNK-specific phosphorylation motif via GPS6.0 kinase-specific phosphorylation site prediction (https://gps.biocuckoo.cn/online.php) (Chen et al. 2023). Statistical analysis was performed with GraphPad Prism 9.0.

### Chlorophyll fluorescence analysis

Chlorophyll fluorescence imaging analysis was conducted via a plant pathological phenotype system called PathoScreen^ProBlu^ (PhenoVation, Wageningen, Netherlands). The chlorophyll fluorescence parameter (Fv/Fm) was used to quantify the severity of late blight. A low Fv/Fm value indicates a more severe disease state. I, II, III, IV, and V indicate ranges of 0.8–0.64, 0.63–0.48, 0.47–0.32, 0.31–0.16, and 0.15–0, respectively.

## Supplementary Information

Supplementary Material 1: Fig. 1. Structure and evolutionary analysis of the Avr2 family effectors. a, The phylogenetic tree of Avr2 family members and the protein structure of Avr2/AL3. b, Amino acid sequence alignment of Avr2 family members.

Supplementary Material 2: Fig. 2. Virulence and subcellular localization analysis of effectors. a, Both Avr2 and AL3 induce HR in the leaves of *Rpi-mcq1* transgenic *N. benthamiana*. The photo was taken five days after the infiltration, *n* = 5. 1. Avr2; 2. PITG_05121; 3. PITG_19617; 4. PITG_07500; 5. PITG_07499; 6. PITG_08278; 7. PITG_15972; 8. PITG_06077; 9. PITG_21949; 10. AL3; 11.PITG_13940; 12. PITG_13936; 13. PEXRD11; 14. MgCl_2_. b, The virulence of Avr2 family members. Images were photographed at 2–4 dpi. c, The biomass of *P. infestans*, t-test (*P < 0.05; **P < 0.01; ***P < 0.001; ****P < 0.0001). d, Subcellular localization of Avr2 and AL3. e–f, Quantitative statistics and the expression levels of cLUC/nLUC fusion genes corresponding to Fig. 1f, t-test (*P* < 0.05).

Supplementary Material 3: Fig. 3. The recognition of Rpi-mcq1-AL3 is independent of StBSLs. a-c, There were no direct interactions between StBSL1/Avr2/AL3 and R2/Rpi-mcq1, scale bars, 20 µm. d, The original picture of Fig. 1h. e–f, The recognition of Rpi-mcq1-AL3 was independent of StBSLs. The efficiency of StBSLs silencing was verified by qRT-PCR. The HR phenotype was observed 5 days after infiltration.

Supplementary Material 4: Fig. 4. ATP6V1C1 is conserved across plant species. a-b, The phylogenetic tree and amino acid sequence alignment of ATP6V1C1 across plant species.

Supplementary Material 5: Fig. 5. StATP6V1C1 positive regulates potato late blight resistance. a, There was no interaction observed between StATP6V1C1 and R2/Rpi-mcq1 in the Y2H assay. b-c, Quantitative statistics and the expression levels of cLUC/nLUC fusion genes corresponding to Fig. 2e, t-test (*P* < 0.05). d, The original image of Fig. 2g. e, The expression level of *StPR5* is higher in the *StATP6V1C1*-OE transgenic lines compared to the ‘Désirée’ variety. f-g, Transient expression of* StATP6V1C1* enhanced potato resistance to late blight. After 1 day of transient expression, the leaves were inoculated with EC1, EV (pCXSN-Myc), OE:*StATP6V1C1* (pCXSN-*StATP6V1C1*-Myc). The percentages of each disease category were recorded at 4 dpi, *n* = 30. h and i, Avr2 and AL3 bidirectionally manipulated intracellular pH level of *N. benthamiana* leaves. Transient expression of pTA7001-Avr2 or pTA7001-AL3 two days, 10 μM of dexamethasone (DEX) or ddH_2_O (Mock) was sprayed onto the leaf. Fluorescence intensity image (h) and statistical data of pH values (i) were obtained after 12 h. Scale bars, 30 µm. Data were presented as means, *n* = 30, two-way ANOVA (*P* < 0.05).

Supplementary Material 6: Fig. 6. Relationship between immunity and interacellular pH level, and evolutionary analysis of WNK family proteins. a-c, Intracellular pH influences *N. benthamiana* resistance to late blight. Leaves were pre-infiltrated with gradient pH buffers one day before inoculation. The Fv/Fm value and the biomass of *P. infestans* EC1 were measured at 48 hpi and 72 hpi, respectively. d and e, StATP6V1C1 interacted with StATP6V1G and StATP6V1E. f, The expression levels of the cLUC/nLUC fusion genes corresponding to Fig. 5b. g, Phylogenetic tree analysis of WNK proteins from potato and *Arabidopsis thaliana*.

Supplementary Material 7: Fig. 7. pH shaking enhances the targeting of Avr2/AL3-StBSL1 and influences the plant resistance to late blight. a, The expression levels of cLUC/nLUC fusion genes corresponding to Fig. 6b. b, StATP6V1C1 proteins with point mutations were detected using western blot analysis. The following conditions were employed to induce protein expression prior to purification: IPTG (0.1 mM) and 16℃ for 16 h. c, The biomass of *P. infestans *EC1 in TRV::00 and TRV::*StWNK10* plants. d, The biomass of *P. infestans* EC1 in *StWNK10* overexpression plants. e–g, StATP6V1C1^S261^ phosphorylation positively regulates the potato resistance to late blight.

Supplementary Material 8: Fig. 8. The overexpression of *StATP6V1C1* affected the development of potato compound leaves. e, The growth height, number of leaves, and ratio of abnormal leaves in OE:*StATP6V1C1* plants, *n* = 10, t-test, *P* < 0.01. f, The relative expression levels of BR-related genes in OE:*StATP6V1C1* plants, t-test, *P* < 0.01.

Supplementary Material 9: Fig. 9. The standard curves for cellular pH measurement. a, The standard curve of *N. benthamiana* for cellular pH measurement. b, The standard curve of ‘Désirée’ for cellular pH measurement.

Supplementary Material 10: Fig. 10. Data for intracellular pH measurement using BCECF-AM method. a, The pH value of Fig. 4c. b, The pH value of Fig. 4e. c, The pH value of Fig. 4h. d, The pH value of Fig. 6j. e, The pH value of Fig. 6h. f, The pH value of Fig. 7f.

Supplementary Material 11: Fig. 11. Western blot of total extracts from infiltrated leaf areas imaged by BiFC. a, Western blot analyses of BiFC construct combinations from the same experiments as in Fig. 1d. b, Western blot analyses of Fig. 1e. c, Western blot analyses of Fig. 2c. d, Western blot analyses of Fig. 2d. e, Western blot analyses of Fig. 7b. f, Western blot analyses of Fig. 7d. g, Western blot analyses of Fig. 7e. h, Western blot analyses of Fig. 7f. i and j, Western blot analyses of Fig. S3c.

Supplementary Material 12. The list of primers used in this study.

## Data Availability

Sequence data used in this article can be found in the GenBank data libraries under accession numbers: PITG_22870 (XM_002902940), PITG_23008 (XM_002899560), StATP6V1C1 (XM_006363911), StBSL1 (NM_001318640), StBSL2/3-like (NM_001288309), StATP6V1G (XM_015308645), StATP6V1E (XM_006343836), StWNK10 (XM_006343735), AtWNK8 (AT5G41990).

## Abbreviations

NLRs: Nucleotide-binding leucine-rich repeat receptors
ETS: Effector-triggered susceptibility
ETI: Effector-triggered immunity
PTI: PAMP-triggered immunity
BR: Brassinosteroids
BiFC: Bimolecular fluorescence complementation
Y2H: Yeast two-hybrid
LCI: Luciferase complementation imaging
VIGS: Virus-induced gene silencing
qRT-PCR: Reverse transcription-quantitative polymerase chain reaction
BCECF-AM: 2',7'-bis-(2-carboxyethyl)-5-(and-6)-carboxyfluorescein,acetoxymethyl ester
HR: Hypersensitive response
EV: Empty vector
Con A: Concanamycin A
