# Supplementary figures and images for: Two Phytophthora effectors mitigate plant immunity by manipulating intracellular pH through interaction with V-ATPase in potato
Source: Mol Hortic. 2026 Jan 9;6:4. doi: 10.1186/s43897-025-00184-w (PMC12784495; doi:10.1186/s43897-025-00184-w)

**Figure S1**

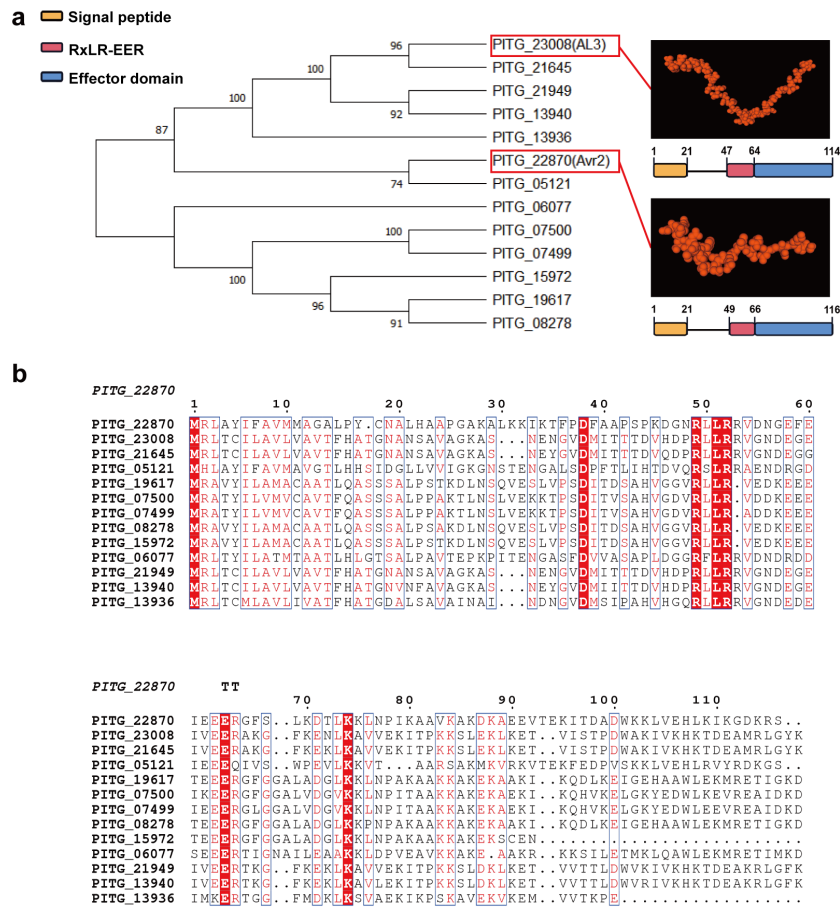

Supplement: Supplementary file 1 — Supplementary Material 1: Fig. 1. Structure and evolutionary analysis of the Avr2 family effectors. a, The phylogenetic tree of Avr2 family members and the protein structure of Avr2/AL3. b, Amino acid sequence alignment of Avr2 family members. [file 43897_2025_184_MOESM1_ESM.pdf]

**Figure S2**

**a**

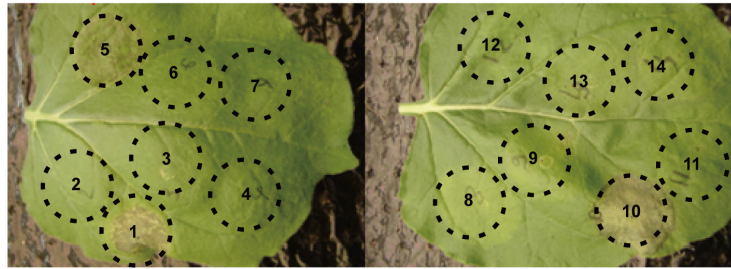

**b**

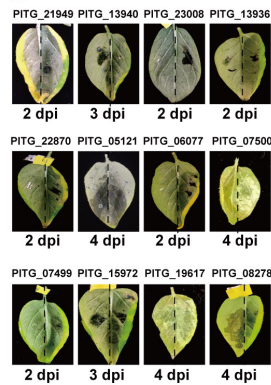

**c**

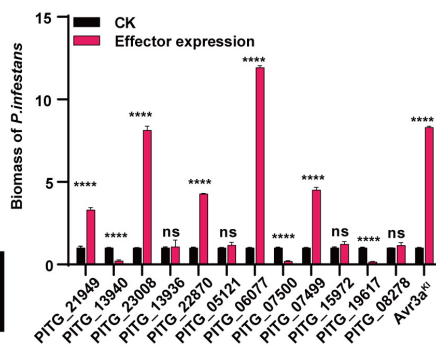

**d**

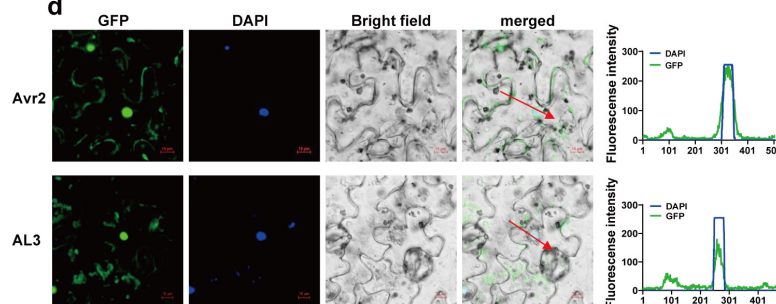

**e**

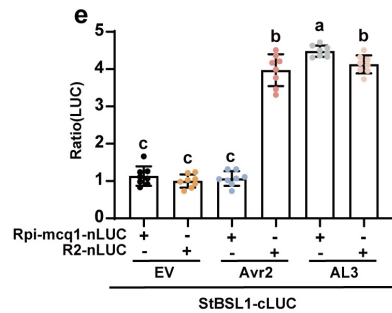

**f**

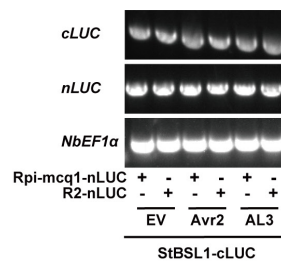

Supplement: Supplementary file 2 — Supplementary Material 2: Fig. 2. Virulence and subcellular localization analysis of effectors. a, Both Avr2 and AL3 induce HR in the leaves of Rpi-mcq1 transgenic N. benthamiana. The photo was taken five days after the infiltration, n = 5. 1. Avr2; 2. PITG_05121; 3. PITG_19617; 4. PITG_07500; 5. PITG_07499; 6. PITG_08278; 7. PITG_15972; 8. PITG_06077; 9. PITG_21949; 10. AL3; 11.PITG_13940; 12. PITG_13936; 13. PEXRD11; 14. MgCl2. b, The virulence of Avr2 family members. Images were photographed at 2–4 dpi. c, The biomass of P. infestans, t-test (*P < 0.05; **P < 0.01; ***P < 0.001; ****P < 0.0001). d, Subcellular localization of Avr2 and AL3. e–f, Quantitative statistics and the expression levels of cLUC/nLUC fusion genes corresponding to Fig. 1f, t-test (P < 0.05). [file 43897_2025_184_MOESM2_ESM.pdf]

**Figure S3**

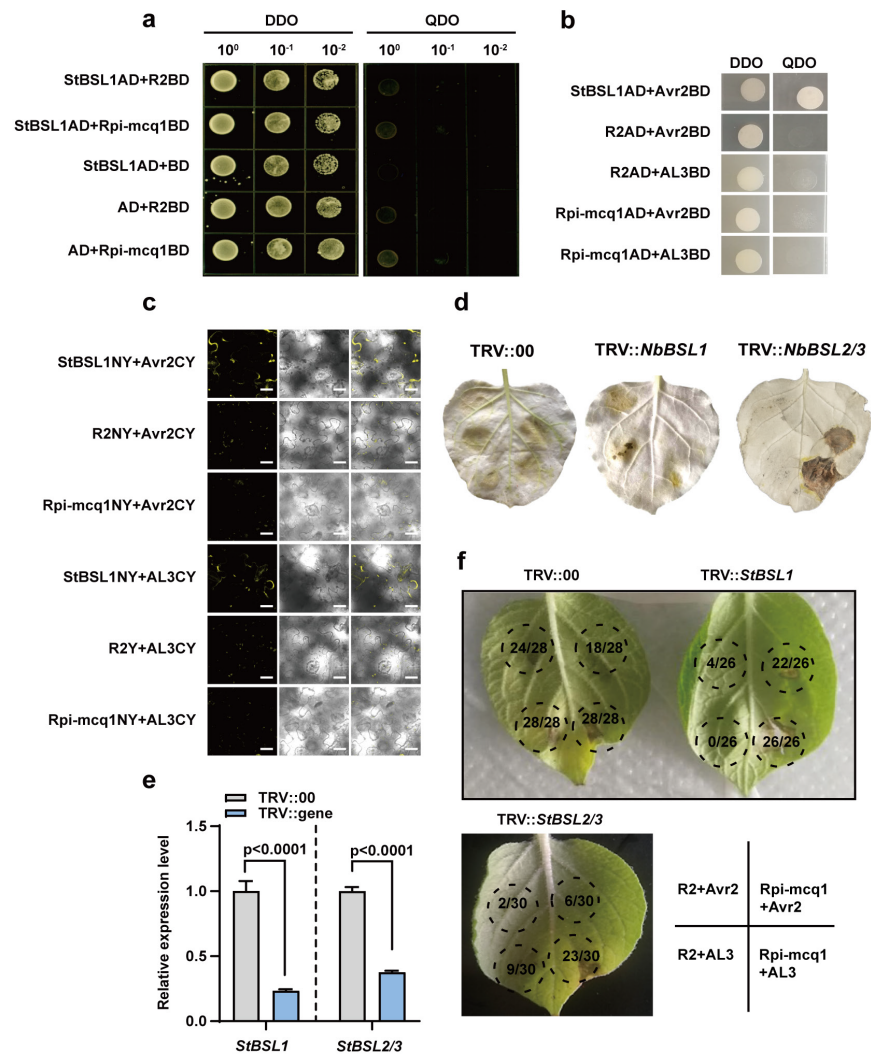

Supplement: Supplementary file 3 — Supplementary Material 3: Fig. 3. The recognition of Rpi-mcq1-AL3 is independent of StBSLs. a-c, There were no direct interactions between StBSL1/Avr2/AL3 and R2/Rpi-mcq1, scale bars, 20 µm. d, The original picture of Fig. 1h. e–f, The recognition of Rpi-mcq1-AL3 was independent of StBSLs. The efficiency of StBSLs silencing was verified by qRT-PCR. The HR phenotype was observed 5 days after infiltration. [file 43897_2025_184_MOESM3_ESM.pdf]

Figure S4

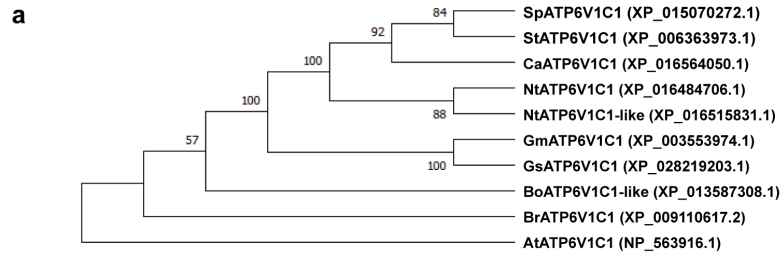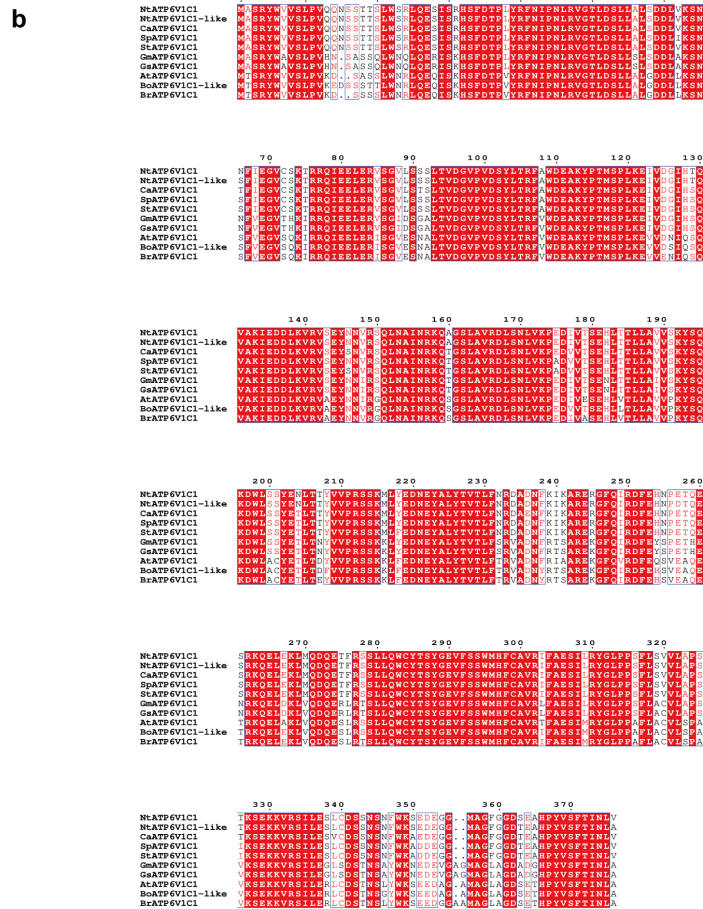

Supplement: Supplementary file 4 — Supplementary Material 4: Fig. 4. ATP6V1C1 is conserved across plant species. a-b, The phylogenetic tree and amino acid sequence alignment of ATP6V1C1 across plant species. [file 43897_2025_184_MOESM4_ESM.pdf]

Figure S5

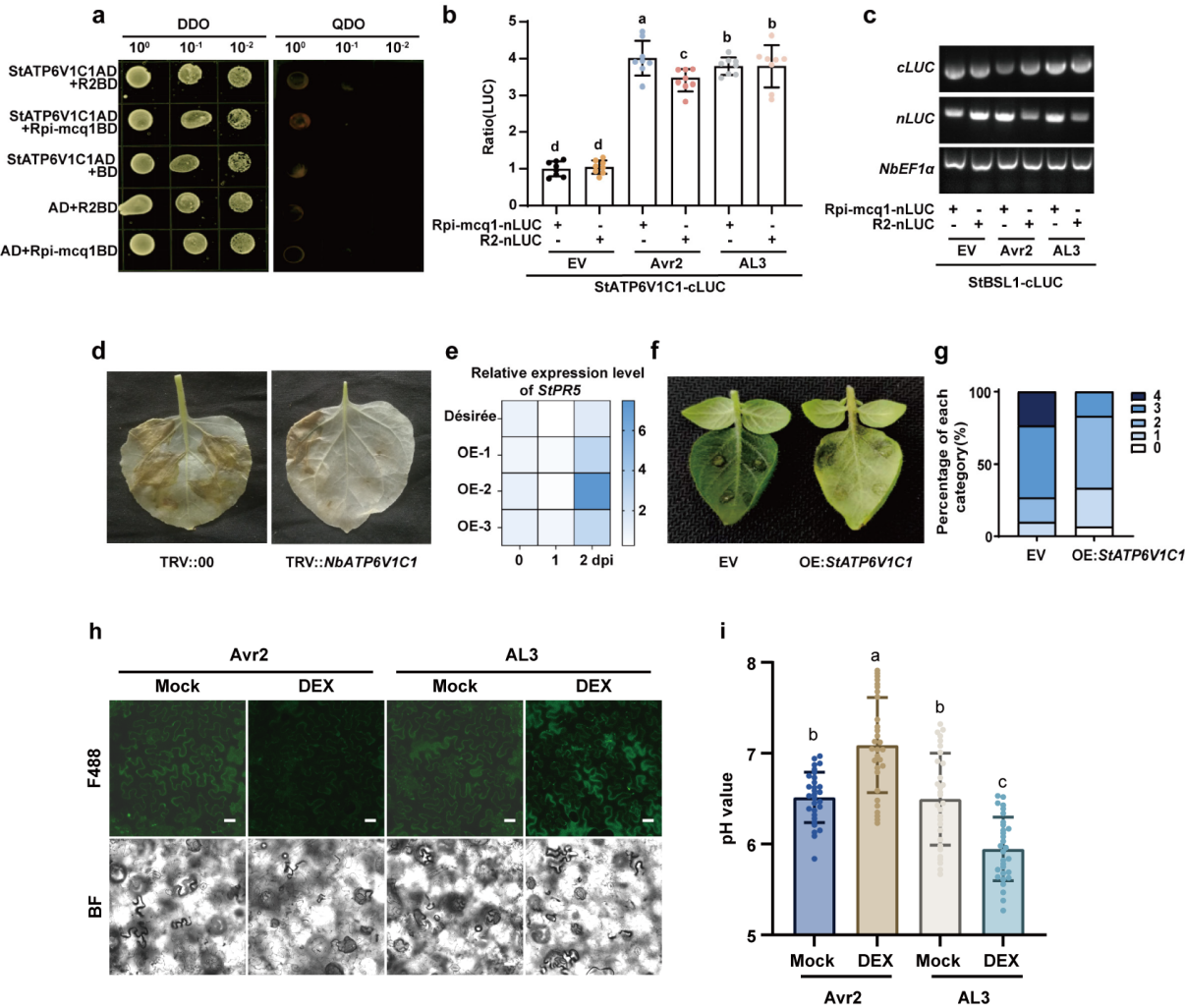

Supplement: Supplementary file 5 — Supplementary Material 5: Fig. 5. StATP6V1C1 positive regulates potato late blight resistance. a, There was no interaction observed between StATP6V1C1 and R2/Rpi-mcq1 in the Y2H assay. b-c, Quantitative statistics and the expression levels of cLUC/nLUC fusion genes corresponding to Fig. 2e, t-test (P < 0.05). d, The original image of Fig. 2g. e, The expression level of StPR5 is higher in the StATP6V1C1-OE transgenic lines compared to the ‘Désirée’ variety. f-g, Transient expression of StATP6V1C1 enhanced potato resistance to late blight. After 1 day of transient expression, the leaves were inoculated with EC1, EV (pCXSN-Myc), OE:StATP6V1C1 (pCXSN-StATP6V1C1-Myc). The percentages of each disease category were recorded at 4 dpi, n = 30. h and i, Avr2 and AL3 bidirectionally manipulated intracellular pH level of N. benthamiana leaves. Transient expression of pTA7001-Avr2 or pTA7001-AL3 two days, 10 μM of dexamethasone (DEX) or ddH2O (Mock) was sprayed onto the leaf. Fluorescence intensity image (h) and statistical data of pH values (i) were obtained after 12 h. Scale bars, 30 µm. Data were presented as means, n = 30, two-way ANOVA (P < 0.05). [file 43897_2025_184_MOESM5_ESM.pdf]

Figure S6

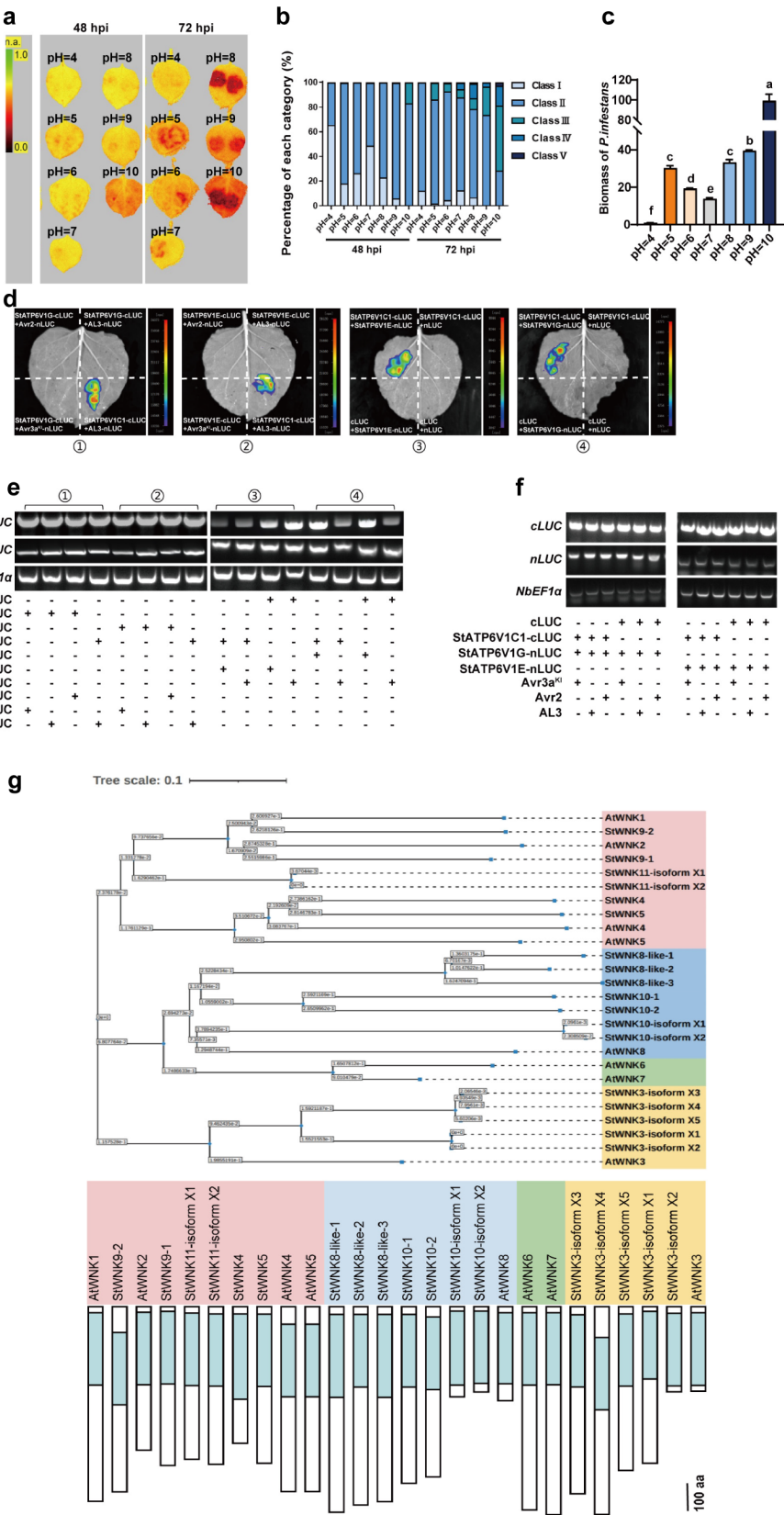

Supplement: Supplementary file 6 — Supplementary Material 6: Fig. 6. Relationship between immunity and interacellular pH level, and evolutionary analysis of WNK family proteins. a-c, Intracellular pH influences N. benthamiana resistance to late blight. Leaves were pre-infiltrated with gradient pH buffers one day before inoculation. The Fv/Fm value and the biomass of P. infestans EC1 were measured at 48 hpi and 72 hpi, respectively. d and e, StATP6V1C1 interacted with StATP6V1G and StATP6V1E. f, The expression levels of the cLUC/nLUC fusion genes corresponding to Fig. 5b. g, Phylogenetic tree analysis of WNK proteins from potato and Arabidopsis thaliana. [file 43897_2025_184_MOESM6_ESM.pdf]

Figure S7

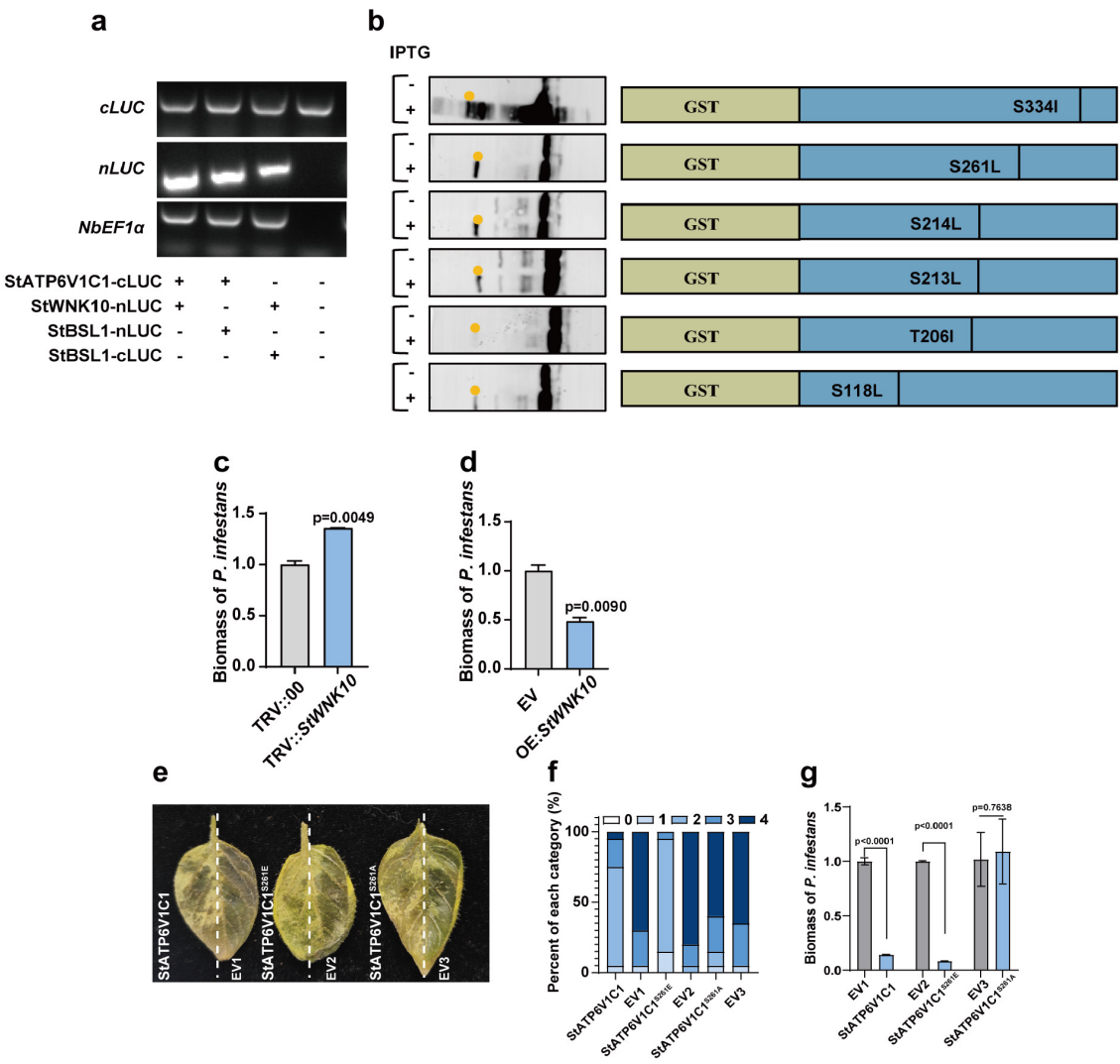

Supplement: Supplementary file 7 — Supplementary Material 7: Fig. 7. pH shaking enhances the targeting of Avr2/AL3-StBSL1 and influences the plant resistance to late blight. a, The expression levels of cLUC/nLUC fusion genes corresponding to Fig. 6b. b, StATP6V1C1 proteins with point mutations were detected using western blot analysis. The following conditions were employed to induce protein expression prior to purification: IPTG (0.1 mM) and 16℃ for 16 h. c, The biomass of P. infestans EC1 in TRV::00 and TRV::StWNK10 plants. d, The biomass of P. infestans EC1 in StWNK10 overexpression plants. e–g, StATP6V1C1S261 phosphorylation positively regulates the potato resistance to late blight. [file 43897_2025_184_MOESM7_ESM.pdf]

**Figure S8**

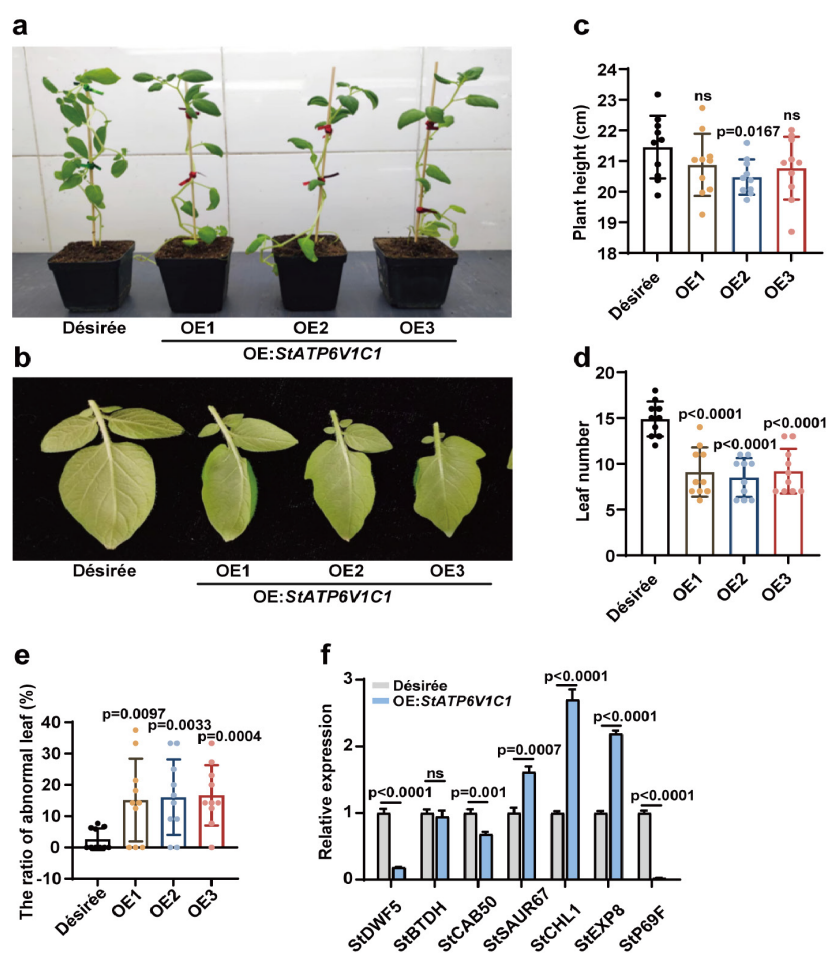

Supplement: Supplementary file 8 — Supplementary Material 8: Fig. 8. The overexpression of StATP6V1C1 affected the development of potato compound leaves. e, The growth height, number of leaves, and ratio of abnormal leaves in OE:StATP6V1C1 plants, n = 10, t-test, P < 0.01. f, The relative expression levels of BR-related genes in OE:StATP6V1C1 plants, t-test, P < 0.01. [file 43897_2025_184_MOESM8_ESM.pdf]

**Figure S9**

**a**

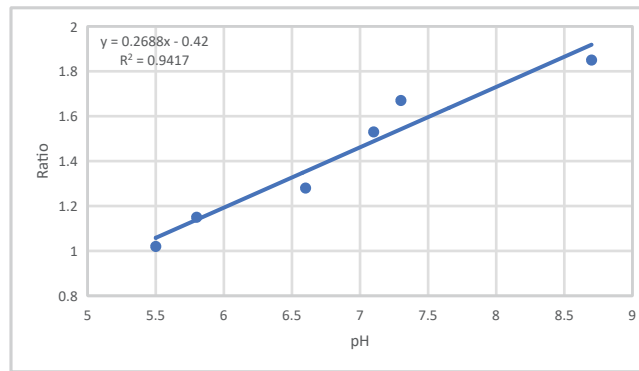

**b**

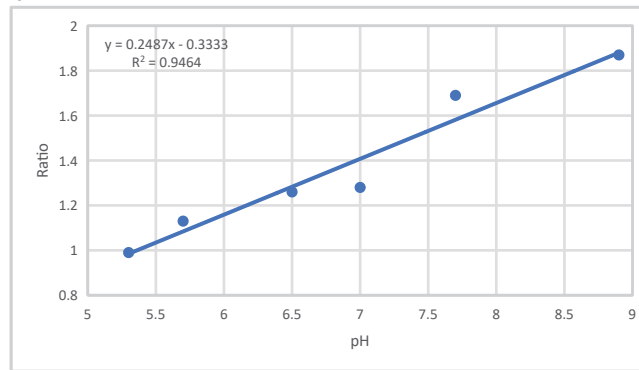

Supplement: Supplementary file 9 — Supplementary Material 9: Fig. 9. The standard curves for cellular pH measurement. a, The standard curve of N. benthamiana for cellular pH measurement. b, The standard curve of ‘Désirée’ for cellular pH measurement. [file 43897_2025_184_MOESM9_ESM.pdf]

Figure S10

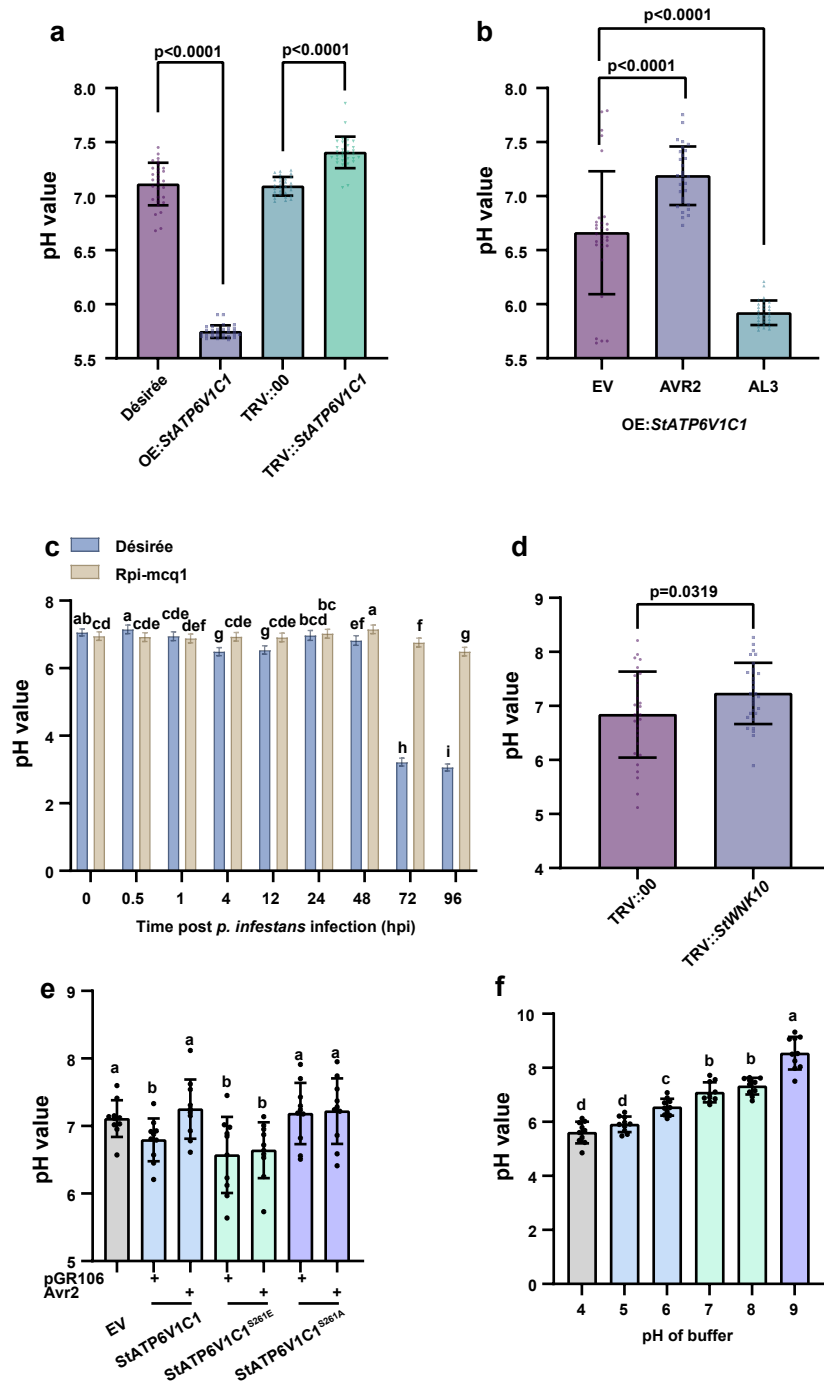

Supplement: Supplementary file 10 — Supplementary Material 10: Fig. 10. Data for intracellular pH measurement using BCECF-AM method. a, The pH value of Fig. 4c. b, The pH value of Fig. 4e. c, The pH value of Fig. 4h. d, The pH value of Fig. 6j. e, The pH value of Fig. 6h. f, The pH value of Fig. 7f. [file 43897_2025_184_MOESM10_ESM.pdf]

Figure S11

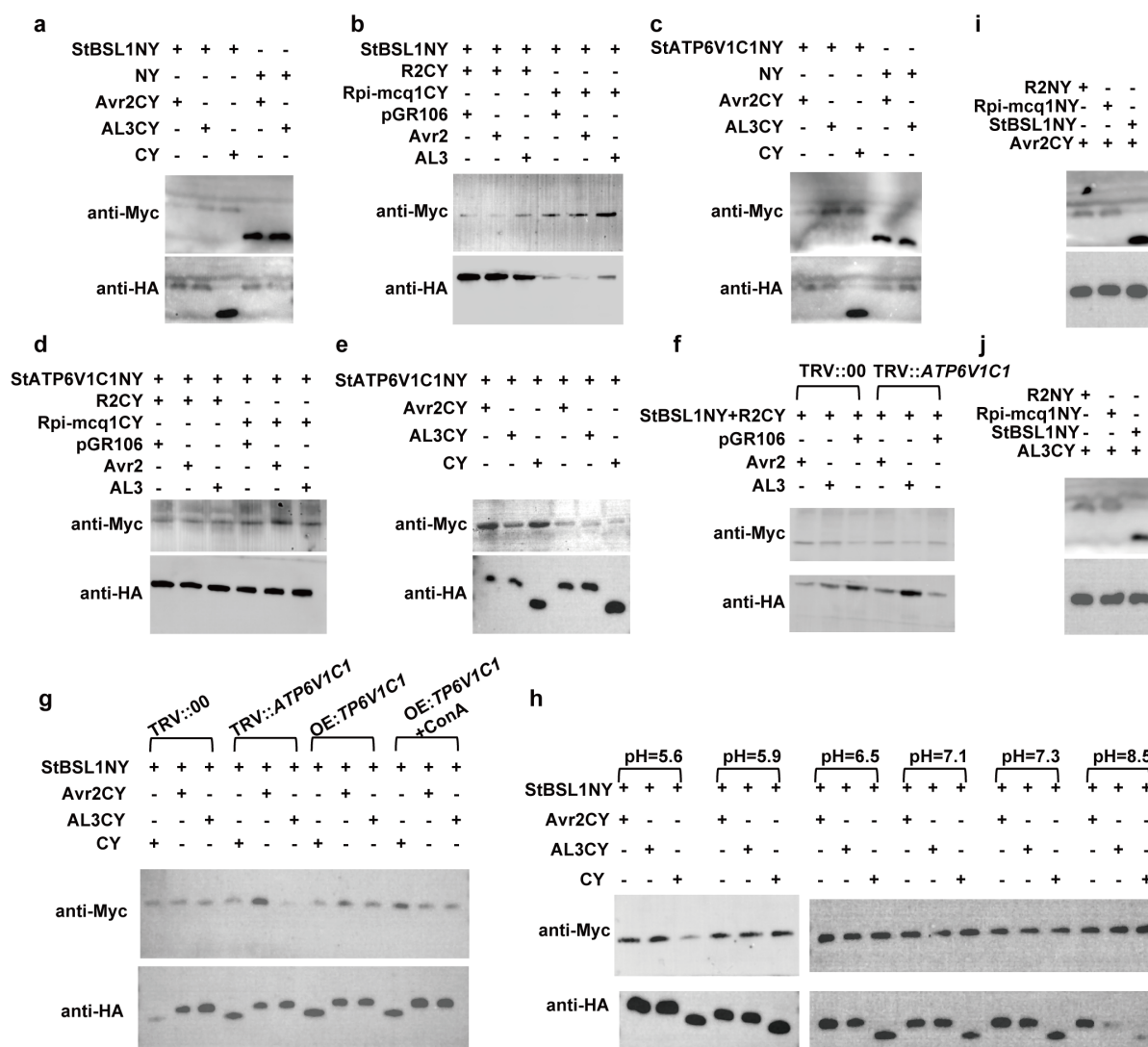

Supplement: Supplementary file 11 — Supplementary Material 11: Fig. 11. Western blot of total extracts from infiltrated leaf areas imaged by BiFC. a, Western blot analyses of BiFC construct combinations from the same experiments as in Fig. 1d. b, Western blot analyses of Fig. 1e. c, Western blot analyses of Fig. 2c. d, Western blot analyses of Fig. 2d. e, Western blot analyses of Fig. 7b. f, Western blot analyses of Fig. 7d. g, Western blot analyses of Fig. 7e. h, Western blot analyses of Fig. 7f. i and j, Western blot analyses of Fig. S3c. [file 43897_2025_184_MOESM11_ESM.pdf]
